# Precision Molecular Engineering of Alternating Donor–Acceptor Cycloparaphenylenes: Multidimensional Optoelectronic Response and Chirality Modulation via Polarization-Driven Charge Transfer

**DOI:** 10.3390/molecules30153127

**Published:** 2025-07-25

**Authors:** Danmei Zhu, Xinwen Gai, Yi Zou, Ying Jin, Jingang Wang

**Affiliations:** College of Science, Liaoning Petrochemical University, Fushun 113001, China; zhudanmei@lnpu.edu.cn (D.Z.);

**Keywords:** alternating donor–acceptor, cycloparaphenylene derivatives, photophysical responses, chiroptical properties, density functional theory

## Abstract

In this study, three alternating donor–acceptor (D–A) type [12]cycloparaphenylene ([12]CPP) derivatives ([12]CPP 1a, 2a, and 3a) were designed through precise molecular engineering, and their multidimensional photophysical responses and chiroptical properties were systematically investigated. The effects of the alternating D–A architecture on electronic structure, excited-state dynamics, and optical behavior were elucidated through density functional theory (DFT) and time-dependent DFT (TD-DFT) calculations. The results show that the alternating D–A design significantly reduced the HOMO–LUMO energy gap (e.g., 3.11 eV for [12]CPP 2a), enhanced charge transfer characteristics, and induced pronounced red-shifted absorption. The introduction of an imide-based acceptor ([12]CPP 2a) further strengthened the electron push-pull interaction, exhibiting superior performance in two-photon absorption, while the symmetrically multifunctionalized structure ([12]CPP 3a) predominantly exhibited localized excitation with the highest absorption intensity but lacked charge transfer features. Chiral analysis reveals that the alternating D–A architecture modulated the distribution of chiral signals, with [12]CPP 1a displaying a strong Cotton effect in the low-wavelength region. These findings not only provide a theoretical basis for the molecular design of functionalized CPP derivatives, but also lay a solid theoretical foundation for expanding their application potential in optoelectronic devices and chiral functional materials.

## 1. Introduction

π-conjugated cyclic systems featuring alternating donor–acceptor (D–A) units have garnered significant attention due to their exceptional photophysical properties, including narrow HOMO–LUMO energy gaps, tunable fluorescence, and nonlinear optical responses. These attributes highlight their potential for use in organic electronics, photonics, and the development of advanced optical materials [1,2,3]. [N]Cycloparaphenylenes ([N]CPPs), a class of macrocyclic π-conjugated compounds composed of para-linked benzene rings, have emerged as key molecular platforms in materials science and chemistry, especially following the pioneering synthesis of [9]-, [12]-, and [18]CPPs by the research groups of Jasti in 2008 [4]. Their unique cyclic conjugation and superior optoelectronic performance have significantly advanced the understanding of π-conjugated molecular systems and opened new possibilities for the design of functional materials [5,6,7].

Recent efforts have focused on enhancing the chemical and functional versatility of CPPs through strategic functionalization. For instance, the incorporation of carborane moieties have enabled the synthesis of macrocyclic luminophores with high quantum efficiency, exhibiting excellent fluorescence in both solution and solid states, which holds promise for bioimaging and solid-state luminescent materials [8]. Furthermore, Jiang Xiqun et al. demonstrated the self-assembly behavior of non-planar CPPs in solution, forming two-dimensional crystalline films and crystalline vesicles with remarkable morphological control and optoelectronic properties [9]. In another breakthrough, perfluorinated cycloparaphenylenes (PFCPPs) were synthesized via nickel-mediated reductive elimination. The introduction of fluorine atoms promoted unique tubular stacking patterns, enhanced low-temperature fluorescence, and broadened the HOMO–LUMO bandgaps, suggesting promising applications in carbon-based nanomaterials and organic semiconductors [10].

To further optimize the optoelectronic properties of CPPs, alternating D–A cyclic π-conjugated compounds have recently become a focal point. Compared to symmetrically functionalized CPPs, alternating D–A configurations make use of strong electron push–pull effects to significantly increase molecular polarization, thereby providing a new paradigm for modulating exciton behavior and photophysical responses [11]. These compounds retain the cyclic conjugation of CPPs while substantially reducing HOMO–LUMO energy gaps and exhibiting positive solvatofluorochromism. For example, Shuhei Nishigaki et al. [12] synthesized alternating D–A [12]CPPs and [16]CPPs via rhodium-catalyzed intermolecular cross-cyclotrimerization. Subsequent functionalization markedly improved their optical and electrical performance, with theoretical calculations elucidating the molecular basis of their superior optoelectronic properties, thereby guiding the design of high-efficiency materials [12,13].

Building on this foundation, our work focuses on the theoretical investigation of alternating D–A [12]CPP derivatives, particularly those incorporating imide-based acceptors. By analyzing one-photon absorption (OPA) [14,15], two-photon absorption (TPA) [16,17], and chiroptical properties [18,19], in combination with DFT [20] and TD-DFT [21,22,23] calculations, we aim to elucidate the intrinsic relationships between molecular structure and photophysical behavior. Our findings provide critical theoretical insights for advancing the design of high-performance CPP-based materials and expanding the frontiers of CPP chemistry.

## 2. Results

### 2.1. Molecular Structures

**Figure 1:**
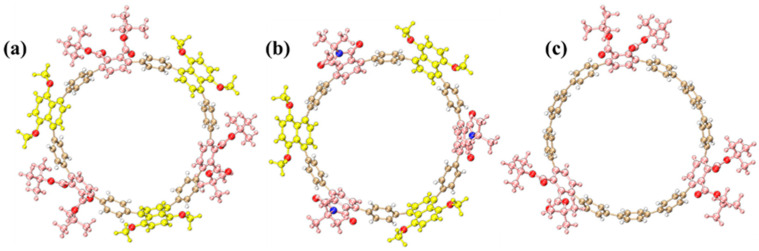
Schematic diagrams of the molecular structures of donor–acceptor nanorings [12]CPP 1a (**a**), [12]CPP 2a (**b**), and the polyfunctionalized CPP structure [12]CPP hexacarboxylate [12]CPP 3a (**c**). Here, the golden spheres represent carbon atoms (C), white represents hydrogen atoms (H), red represents oxygen atoms (O), and blue represents nitrogen atoms (N). In the molecular structures, the strong donor moieties are highlighted in yellow, and the strong acceptor moieties are highlighted in pink.

The optimized molecular structures of three [12]cycloparaphenylene ([12]CPP) derivatives exhibit distinct structural configurations (Figure 1). The alternating donor–acceptor (D–A) [12]CPP ([12]CPP 1a) comprises 1,4-dimethoxynaphthalene (donor) and phthalate (acceptor) units alternately arranged along the cyclic CPP backbone, forming a nanoring with pronounced electron push-pull characteristics (Figure 1a). This configuration facilitates efficient intramolecular charge transfer, substantially reducing the HOMO–LUMO energy gap and imparting unique photophysical properties.

For [12]CPP 2a, the acceptor unit is replaced with a stronger electron-withdrawing phthalimide group via imidation, leading to a more polarized D–A configuration (Figure 1b). The introduction of the imide moiety further amplifies the acceptor’s electron affinity, leading to a narrower HOMO–LUMO gap. As a control, the symmetrically functionalized [12]CPP hexacarboxylate ([12]CPP 3a) features three phthalate groups uniformly distributed on the CPP ring, devoid of alternating D–A motifs (Figure 1c). Structural optimizations confirm that all derivatives retain the cyclic π-conjugated framework of CPPs (Figure 1), while the introduced functional groups modulate both electronic environments and conformational dynamics, thereby critically influencing their photophysical behaviors. The ChemDraw 19.1 schemes of the three structures are shown in Figure S1, and the corresponding atomic coordinates are listed in Tables S2–S4.

As a key parameter for evaluating the structural characteristics of cyclic π-conjugated molecules, the inner diameter data of [12]CPP 1a, [12]CPP 2a, and [12]CPP 3a are listed in Table 1. For these three differently structured [12]CPP derivatives, the inner diameter is defined as the distance between the centroids of two para-positioned benzene rings within the molecule. Schematic diagrams defining the inner diameter are shown on the right-hand side of Table 1 and Figure S2. A comparison with the structural parameters of the parent [12]CPP and the reduced-state [12]CPP [24] shows that their distortion parameters (D.P.) are in good agreement with that of [12]CPP (D.P. = 1.03), fluctuating within a narrow range of 0.01–0.02, and are significantly reduced by approximately 0.2 compared to the D.P. value of the reduced-state [12]CPP. This indicates that the molecular skeletons maintain planar conjugated characteristics similar to those of the parent molecule. The increased inner diameter of [12]CPP 1a suggests that the introduction of 1,4-dimethoxynaphthalene (donor) and phthalate (acceptor) leads to an expansion of the [12]CPP structure’s inner diameter.

As shown in Figure S3, the dihedral angle is defined as the angle between the planes of two para-substituted benzene rings on the [12]CPP backbone. The dihedral angle ranges of [12]CPP 1a and [12]CPP 2a are relatively narrow with concentrated values, which implies that the interactions within their donor–acceptor structures may render the arrangement of benzene rings more regular. [12]CPP 3a, as a single-receptor structure, possesses the broadest dihedral angle range (99.96–152.63°), revealing a more diverse spatial orientation of benzene rings. This is likely due to the absence of donor interactions, which results in less constraint on the arrangement of the benzene rings. This discrepancy reflects the impact of molecular design (donor–acceptor vs. single–receptor) on the spatial configuration of benzene rings, providing crucial structural insights for a deep understanding of intermolecular interaction mechanisms and structure–function relationships.

### 2.2. Density of States and HOMO–LUMO Energy Level Analysis

**Figure 2:**
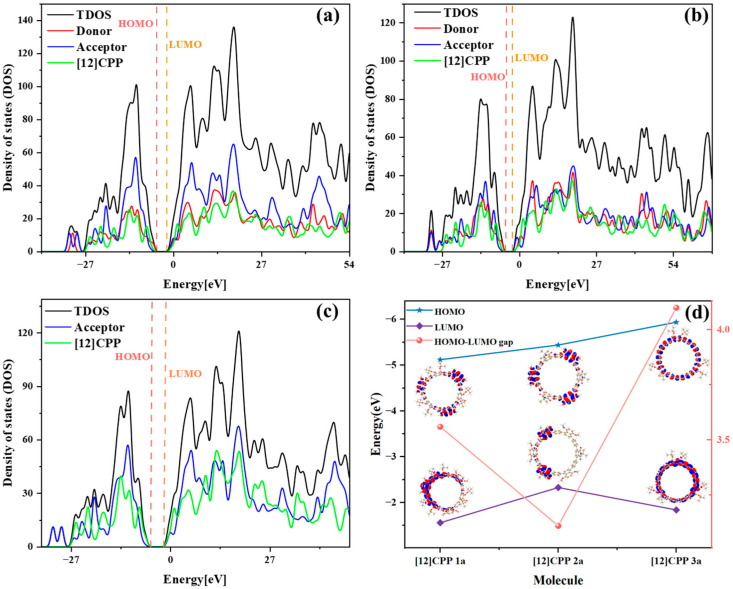
Density of states (DOS) plots for [12]CPP 1a (**a**), [12]CPP 2a (**b**), and [12]CPP 3a (**c**), HOMO–LUMO energy level diagrams, and isosurface plots (**d**) of HOMO and LUMO for [12]CPP 1a, [12]CPP 2a, and [12]CPP 3a; the red isosurfaces represent electrons, while the blue isosurfaces represent holes. The isosurface value is set to 0.02.

Figure 2a–c illustrate the density of states (DOS) plots for [12]CPP 1a, [12]CPP 2a, and [12]CPP 3a, respectively, including a detailed analysis of the contributions from the donor, acceptor, and [12]CPP host to the molecular DOS. The DOS profiles of the three structures reveal the pronounced influence of donor and acceptor incorporation on their electronic structures. For [12]CPP 1a, the acceptor contributes more significantly than both the donor and the CPP host, indicating that it dominates the electron distribution and plays a key role in LUMO formation. In [12]CPP 2a, the contributions from the donor, acceptor, and CPP host are relatively balanced, suggesting that the electron distribution in this structure is co-regulated by both donor and acceptor units. The contributions from the donor’s HOMO and the acceptor’s LUMO are both significant, further reducing the HOMO–LUMO energy gap of the molecule. In contrast, the energy gap of [12]CPP 1a is slightly larger (approximately 3.56 eV), while the symmetric structure of [12]CPP 3a, due to the absence of donor–acceptor interactions, exhibits the largest HOMO–LUMO gap (approximately 4.09 eV). HOMO–LUMO isosurface diagrams reveal that the HOMO of [12]CPP 1a is localized on the 1,4-dimethoxynaphthalene, and the LUMO is predominantly located on the phthalate ester, with minor distributions elsewhere. In the case of [12]CPP 2a, the HOMO is entirely concentrated on the 1,4-dimethoxynaphthalene, and the LUMO is entirely concentrated on the phthalimide, demonstrating a pronounced donor–acceptor polarization effect. For [12]CPP 3a, both the HOMO and LUMO are primarily localized on the main skeleton, with no energy degeneracy observed between them. Additionally, frontier orbital energy diagrams were plotted for the three structures (Figure S4), further illustrating the energy differences between occupied and unoccupied orbitals among them. The results reveal that no orbital degeneracy occurred in these three structures for these orbitals. This non-degenerate orbital distribution characteristic indicates that the intramolecular electronic transitions exhibit well-defined directionality. This distribution difference further elucidates the significant influence of functional groups on electron distribution and photophysical properties.

### 2.3. OPA Analysis

**Figure 3:**
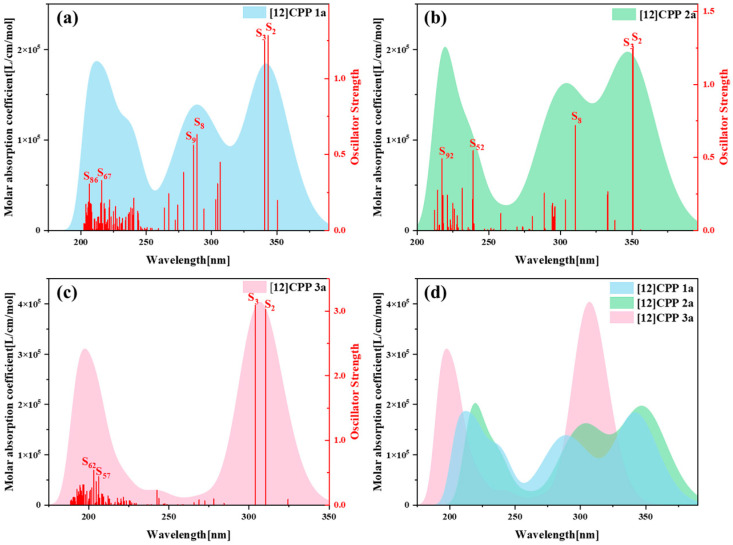
One-photon absorption (OPA) spectra of [12]CPP 1a (**a**), [12]CPP 2a (**b**), and [12]CPP 3a (**c**), and their combined spectrum (**d**).

Figure 3 presents the one-photon absorption spectra of [12]CPP 1a, [12]CPP 2a, and [12]CPP 3a calculated under the chloroform implicit solvent model, revealing significant differences in their absorption intensities and peak positions. Figure S5 displays the comparison between the computational results and experimental data for the [12]CPP 1a and [12]CPP 2a structures. The experimental absorption spectra (c,d) in Figure S5 are adapted from Ref. [12]. The strong agreement between the experimental and calculated values confirms the accuracy of the theoretical calculations. The spectra indicate that [12]CPP 3a exhibits a higher molar absorption coefficient. Compared to [12]CPP 3a, the absorption spectrum of [12]CPP 1a displays a distinct redshift, primarily attributed to the molecular polarization effect induced by the introduction of an alternating donor–acceptor structure. Furthermore, [12]CPP 2a exhibits an even greater redshift compared to [12]CPP 1a, which is attributed to the strong electron-withdrawing nature of the imide acceptor that significantly lowers the LUMO energy level, thereby reducing the HOMO–LUMO gap and decreasing the transition energy. In the three structures, the excited states making the largest contributions to the absorption peaks are S_2_ and S_3_, among which S_2_ and S_3_ in [12]CPP 2a represent degenerate excited states.

**Figure 4:**
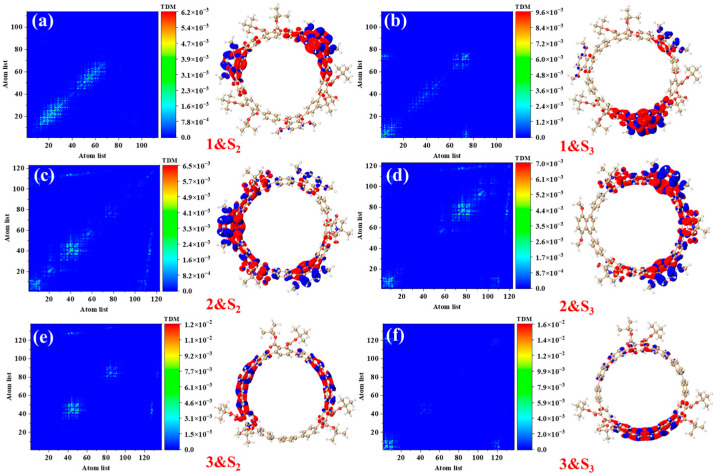
Transition density matrix (TDM) plots and electron–hole density plots of [12]CPP 1a under S_2_ (**a**) and S_3_ (**b**) states, [12]CPP 2a under S_2_ (**c**) and S_3_ (**d**) states, and [12]CPP 3a under S_2_ (**e**) and S_3_ (**f**) states. In the electron–hole density plots, red represents electrons and blue represents holes, with an isosurface value of 0.05.

Figure 4 presents the transition density matrices (TDM) and electron–hole density distribution maps for [12]CPP 1a, [12]CPP 2a, and [12]CPP 3a in the S_2_ and S_3_ excited states, offering a visual comparison of their one-photon absorption characteristics. In the TDM maps, electrons and holes in [12]CPP 1a and 2a exhibit pronounced localization features. The transition density of [12]CPP 1a is predominantly concentrated in the donor region, while that of [12]CPP 2a is distributed across both the donor and acceptor regions, reflecting strong electronic coupling between the donor–acceptor units. In the electron–hole density maps, both electrons and holes in [12]CPP 1a and [12]CPP 2a are mainly localized in the donor region. A clear separation is observed: electrons are concentrated on the CPP ring, while holes are localized on the upper functional groups, suggesting significant electron–hole separation. The [12]CPP 2a structure also exhibits some electron–hole density in the donor region. The excitation types of [12]CPP 1a and [12]CPP 2a are characterized by a combination of charge transfer and localized excitation. Moreover, in the S_2_ and S_3_ states, electron–hole distributions are localized in distinct acceptor regions, suggesting that the donor–acceptor coupling mode depends on the excitation state. For [12]CPP 3a, due to the absence of a donor–acceptor alternating structure, its TDM map exhibits a relatively uniform transition distribution, suggesting that the electronic transitions are primarily confined within the [12]CPP host structure. The electron–hole density maps reveal that, in the S_2_ and S_3_ excited states, the electron and hole densities are uniformly distributed across the [12]CPP ring and exhibit a certain spatial complementarity, lacking significant polarization characteristics.

Based on a systematic analysis of key excitation parameters (D/Sr/H/t indices, Ecoul, and HDI/EDI), the electronic excitation characteristics of [12]CPP derivatives exhibit a clear configuration-dependent behavior, as shown in Table 2. The D index measures the spatial distance between the centroids of the electron and the hole in the excited state; a larger D index indicates greater spatial separation, typically reflecting stronger charge transfer (CT) character. The H index describes the overall average spatial distribution breadth of the electron and hole, while the t index specifically quantifies the degree of electron–hole separation and serves as a key parameter for evaluating exciton dissociation. The Sr index represents the geometric average overlap between the electron and hole distributions, where lower Sr values correspond to weaker spatial overlap. In addition, the hole delocalization index (HDI) and electron delocalization index (EDI) provide a quantitative measure of the degree of delocalization of the hole and electron in the excited state. [12]CPP 2a exhibits partial CT characteristics in its electronic excitation. The relatively large D index suggests pronounced spatial separation between electron and hole distributions. However, its negative t index indicates that localized excitation (LE) features are also significant. Thus, [12]CPP 2a can be classified as a CT-LE hybrid excitation mode. In contrast, [12]CPP 3a has the smallest D value and the most negative Coulomb interaction energy (Ecoul), indicating a stronger exciton binding and a predominantly localized excitation nature. The Sr also supports this observation: the electron–hole overlap in [12]CPP 2a is lower than that in 1a and 3a under both excited states. [12]CPP 3a shows distinct LE features, characterized by the smallest D value and the highest Sr value, implying that the electronic transitions are mainly confined within the CPP framework. [12]CPP 1a displays intermediate values across all parameters, indicating a CT-LE hybrid excitation mechanism as well, though with a dominant LE character. This conclusion is consistent with the transition density distribution patterns shown in Figure 4.

**Figure 5:**
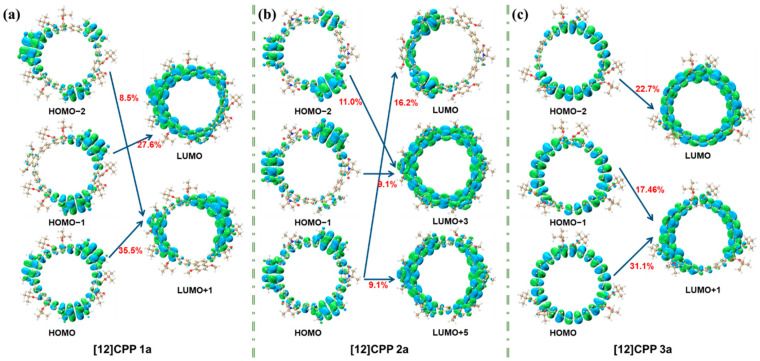
Orbital contributions of [12]CPP 1a (**a**), [12]CPP 2a (**b**), and [12]CPP 3a (**c**) in the S_2_ excited state. The blue isosurfaces represent the negative phase, while the green isosurfaces represent the positive phase.

Quantitative exciton binding energy data effectively characterize Coulomb interaction intensities across different excited states, while orbital contribution analysis (Figure 5) combined with these metrics fully elucidates the donor–acceptor interaction modes. Figure 5 illustrates the orbital contributions of [12]CPP 1a, 2a, and 3a in the S_2_ excited state. For [12]CPP 1a, transitions like HOMO-2→LUMO + 1 (8.5%), HOMO→LUMO + 1 (35.5%), and HOMO-1→LUMO (27.8%) highlight concentrated contributions on specific paths. [12]CPP 2a shows dispersed contributions, reflecting complex orbital interactions. [12]CPP 3a features dominant contributions from HOMO-2→LUMO (22.7%) and HOMO→LUMO + 1 (31.1%). These distinct patterns reveal unique orbital participation in each structure’s electronic excitation, offering microscopic insights into their photophysical properties.

To better elucidate the electron transfer process during electronic excitation, the electron transfer amounts between the donor, acceptor, and [12]CPP fragments in the S_2_ excited state were calculated using the IFCT (Inter-Fragment Charge Transfer) method. The IFCT analysis is based on the contributions of fragments to holes and electrons. In this method, the electron transfer amount from fragment R to fragment S during the electronic excitation process is calculated as follows:

(1) $$Q_{R,S}=\Theta_{R,\mathrm{hole}}\Theta_{S,\mathrm{ele}}$$

In Equation (1), $\Theta_{R,\mathrm{hole}}$ represents the proportion of *R* in the excited electrons, and $\Theta_{S,\mathrm{ele}}$ represents the proportion of *S* in the destination of the electrons.

The net electron transfer amount between two fragments, that is, the difference between the electron transfers in two directions, is shown in Equation (2):

(2) $$P_{S\to R}=Q_{S,R}-Q_{R,S}$$

The net change in the number of electrons of a certain fragment, which is the sum of the net electron transfer amounts between this fragment and all other fragments, is shown in Equation (3).

(3) $$\Delta P_{R}=\sum_{S\neq R}\left(Q_{S,R}-Q_{R,S}\right)$$

Table 3 presents the net electron transfer amounts between different fragments in the three molecules. From the table, it is evident that in [12]CPP 1a, electrons are transferred overall from the donor to other regions, with a net transfer of 0.024 electrons from the donor to the acceptor and 0.089 electrons from the donor to [12]CPP. Additionally, [12]CPP transfers a small number of electrons to the acceptor. Combined with the data in Table 4, the donor loses −0.114 electrons, the acceptor gains 0.027 electrons, and [12]CPP gains 0.087 electrons, indicating that a significant portion of electrons is transferred from the donor to [12]CPP. Table 5 further reveals that, during excitation, electrons in the acceptor and CPP fragments show negligible intra-fragment redistribution, whereas the donor exhibits substantial redistribution (value = 0.56). This indicates that electronic excitation strongly perturbs the donor’s electronic structure, underscoring its pivotal role in the excitation process. In [12]CPP 2a, the net electron transfer from the donor to the acceptor is significantly higher than that from the donor to [12]CPP. Additionally, electrons are transferred from both the donor and CPP fragment to the acceptor, leading to a marked increase in its net charge. The electrons on the donor of [12]CPP also exhibit significant redistribution. In the case of [12]CPP 3a, which lacks a donor, electrons are only transferred from [12]CPP to the acceptor region, and the electrons on [12]CPP undergo substantial redistribution, making the electronic excitation particularly prominent in this part. According to Table 6, the excitation modes of [12]CPP 1a and [12]CPP 2a are characterized by a combination of charge transfer and localized excitation, with [12]CPP 2a exhibiting more pronounced charge transfer characteristics, whereas [12]CPP 3a primarily exhibits localized excitation. This conclusion is entirely consistent with the electron–hole density maps.

Table 7 highlights distinct charge distribution characteristics among the three structures as revealed by NPA analysis [25]. For [12]CPP 1a, the donor moiety and [12]CPP framework exhibit positive charges, while the acceptor group carries a negative charge, indicating that the donor and [12]CPP scaffold act as electron loss centers to facilitate electron transfer to the acceptor. In [12]CPP 2a, the donor bears a higher positive charge than in 1a, while the acceptor exhibits a slightly less negative charge, and the CPP ring carries a lower positive charge. This charge redistribution signifies a more pronounced donor-to-acceptor electron transfer intensity in [12]CPP 2a relative to [12]CPP 1a. For [12]CPP 3a, the absence of a donor group results in opposing charges between the acceptor (−0.099) and [12]CPP framework (0.099), reflecting direct electron transfer between the [12]CPP scaffold and the acceptor moiety. These NPA charge distributions demonstrate that the presence of donor units in 1a and 2a significantly modulates charge allocation compared to 3a, providing critical insights into the electron interaction mechanisms within these systems.

### 2.4. Two-Photon Absorption

**Figure 6:**
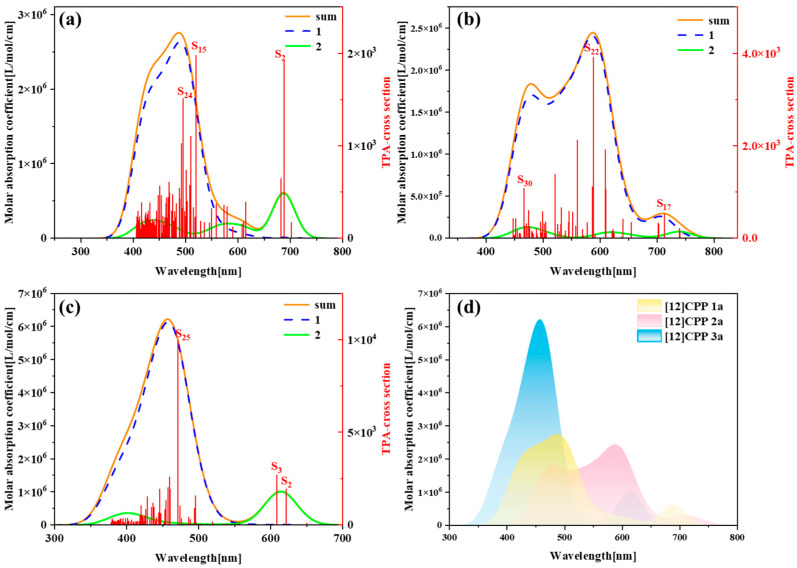
Two-Photon absorption (TPA) spectra of [12]CPP 1a (**a**), [12]CPP 2a (**b**), and [12]CPP 3a (**c**), and their combined spectrum (**d**).

Figure 6 presents the two-photon absorption (TPA) spectra of [12]CPP 1a, [12]CPP 2a, and [12]CPP 3a, along with their comparative overlay, revealing the differences in TPA properties and intramolecular transition mechanisms among the three molecules. TPA is a nonlinear optical process defined as a phenomenon in which a material simultaneously absorbs two low-energy photons (typically near-infrared or infrared light), undergoes excitation transitions and relaxation processes, and subsequently emits fluorescence photons via spontaneous radiation to return to the ground state. The absorption probability is proportional to the square of the incident light intensity. The underlying mechanism involves a two-step excitation pathway mediated by a virtual intermediate state, as illustrated in the Jablonski diagram (Figure S6). It can be observed that the absorption peaks of [12]CPP 2a exhibit a significant redshift, with the absorption peaks located in the range of 400–800 nm. This indicates that the introduction of the imide acceptor effectively lowers the LUMO energy level of the molecule, resulting in a notable reduction in transition energy. Furthermore, the main absorption peaks of [12]CPP 2a originate predominantly from the first-step transition. In contrast, although the absorption peaks of [12]CPP 3a exhibit a significant blueshift, their absorption intensity is the strongest among the three, which is closely related to its symmetric structure and the electronic effects of the hexacarboxylate groups. Simultaneously, in the spectrum of [12]CPP 3a, the small absorption peak on the right side is mainly contributed by the second-step transition, while the main absorption peak is contributed by the first-step transition, indicating that the symmetric structure exhibits different transition modes in the excited-state transitions. The absorption peak distribution and spectral characteristics of [12]CPP 1a exhibit intermediate properties between [12]CPP 2a and [12]CPP 3a: the main absorption peak originates from the first-step transition, while the minor peak on the right side predominantly arises from second-step excited-state transitions, consistent with the dual photon absorption mechanism observed in [12]CPP 1a. This suggests that the donor–acceptor structure of [12]CPP 1a provides a certain degree of polarization, albeit less pronounced than that of [12]CPP 2a.

To more precisely analyze the excitation characteristics in the two-photon transitions, Transition Density Matrix (TDM) and electron–hole density analyses were employed. These results clearly elucidate the regulatory effects of the donor–acceptor structure and symmetry on the two-photon absorption process.

**Figure 7:**
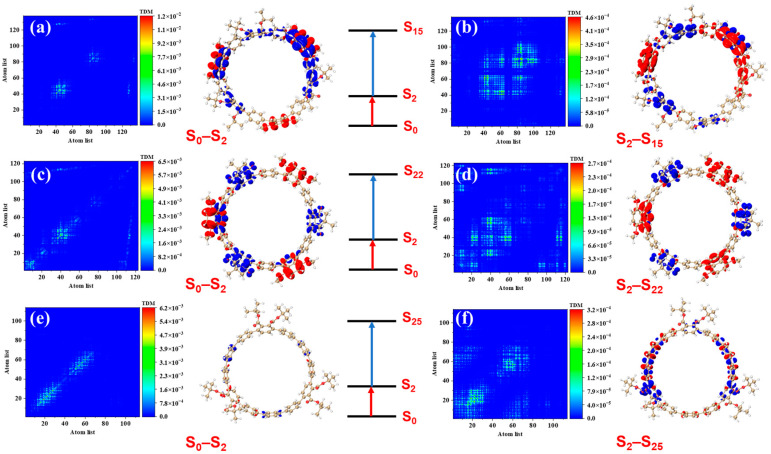
Transition density matrix (TDM) plots and electron–hole density plots of [12]CPP 1a under the two-step transitions of S_0_→S_2_→S_15_ (**a**,**b**), [12]CPP 2a under the two-step transitions of S_0_→S_2_→S_22_ (**c**,**d**), and [12]CPP 3a under the two-step transitions of S_0_→S_2_→S_25_ (**e**,**f**). In the electron–hole density plots, red represents electrons and blue represents holes, with an isosurface value of 0.001.

From Figure 7a,b, it can be observed that [12]CPP 1a exhibits localized excitation and charge transfer characteristics in the S_0_→S_2_ transition, with electrons localized in the lower donor (1,4-dimethoxynaphthalene) and the upper donor’s functional group regions, while holes are concentrated on the [12]CPP ring. In the second-step transition (S_2_→S_15_), the TDM map reveals that the transition density expands from the molecular skeleton to a broader region between the donor and acceptor, with corresponding diffusion of electron and hole distributions. Electrons are concentrated on the donor, while holes are localized on the benzene ring of the acceptor. This transition pattern indicates that the two-photon absorption process of [12]CPP 1a is primarily driven by donor–acceptor interactions, with a gradual transition from localized excitation to charge transfer excitation. In contrast, [12]CPP 2a exhibits more pronounced charge-transfer characteristics in the S_0_→S_2_ transition, with electron density almost entirely localized on the donor (1,4-dimethoxynaphthalene) and hole density on the acceptor (phthalimide), indicating stronger donor–acceptor polarization. In the S_2_→S_22_ transition, the TDM distribution further expands, spanning a larger region of the molecule, and the separation between electrons and holes becomes more significant. This trend indicates that the introduction of the imide acceptor significantly enhances the charge transfer effect of the molecule, enabling [12]CPP 2a to exhibit superior optical performance during the two-photon transition process, as shown in Figure 7c,d. In comparison, [12]CPP 3a contributes very little to the S_0_→S_2_ transition, with minimal electron and hole densities, representing charge transfer excitation. In the S_2_→S_25_ transition, the TDM distribution is confined to the molecular skeleton, with uniform electron and hole distributions, as illustrated in Figure 7e,f.

Building on the above analysis, we delved deeper into the two-photon absorption mechanism by systematically calculating the transition dipole moment matrix elements for the three target molecules during two-photon absorption, with results presented in Table 8. The two steps of the two-photon transition differ significantly in transition probability. The first-step transition, from the ground state to the intermediate state, generally exhibits a higher probability, whereas the second-step transition between excited states is relatively weak. Comparing the computational results across the three molecules, 3a displays particularly striking characteristics: in the first step of the two-photon transition, its transition dipole moment values are the largest, and the corresponding integral value of two-photon excitation is also significantly higher than those of the other molecules, indicating that 3a possesses the strongest two-photon absorption capability. By contrast with 3a, upon introducing donor structures in 1a and 2a, the transition dipole moment values decreased in magnitude, leading to weakened two-photon absorption intensity. Furthermore, in the two-photon absorption processes of 1a and 2a, the transition dipole moments of the first-step transitions dominate, confirming that two-photon absorption is primarily governed by the initial excitation.

### 2.5. Chiral Analysis

**Figure 8:**
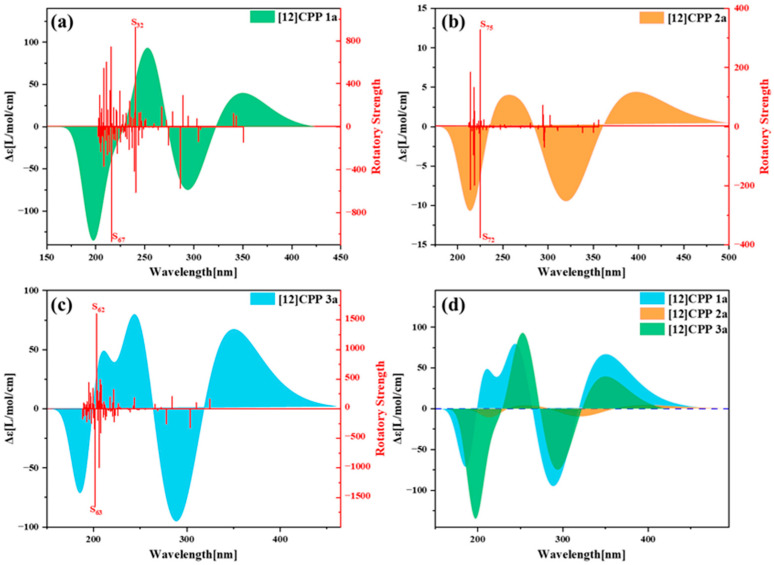
Electronic circular dichroism (ECD) spectra of [12]CPP 1a (**a**), [12]CPP 2a (**b**), and [12]CPP 3a (**c**), as well as the combined spectrum (**d**).

To investigate the influence of donors and acceptors on the molecular chirality, Figure 8 presents the electronic circular dichroism (ECD) spectra of [12]CPP 1a, [12]CPP 2a, and [12]CPP 3a, revealing significant differences in chiroptical activity among the three molecules and their structural regulation mechanisms. Figure S7 depicts the enantiomers of [12]CPP 1a, [12]CPP 2a, and [12]CPP 3a. Figure S8 shows the chiral spectra of the three enantiomers, which are compared with those of [12]CPP 1a, [12]CPP 2a, and [12]CPP 3a. They exhibit optical signals that are mirror-image symmetrical (with opposite absorbance differences). In Figure 8, the spectrum of [12]CPP 2a exhibits a notable redshift, with its chiral signals predominantly located in the longer wavelength region (200–500 nm), although the overall optical rotation intensity is the weakest. In contrast, the chiral activity of [12]CPP 1a is strongest in the lower wavelength region (190–270 nm), and its pronounced Cotton effect indicates that the electronic transitions between the donor and acceptor significantly contribute to the chiroptical activity. Meanwhile, the chiral signals of [12]CPP 3a are concentrated in the higher wavelength region (270–450 nm). Despite the absence of donor–acceptor polarization effects in [12]CPP 3a, its chiral signals are more intense in the higher wavelength region. These results demonstrate that the introduction of donor–acceptor structures plays a regulatory role in the intensity and distribution of chiral signals.

**Figure 9:**
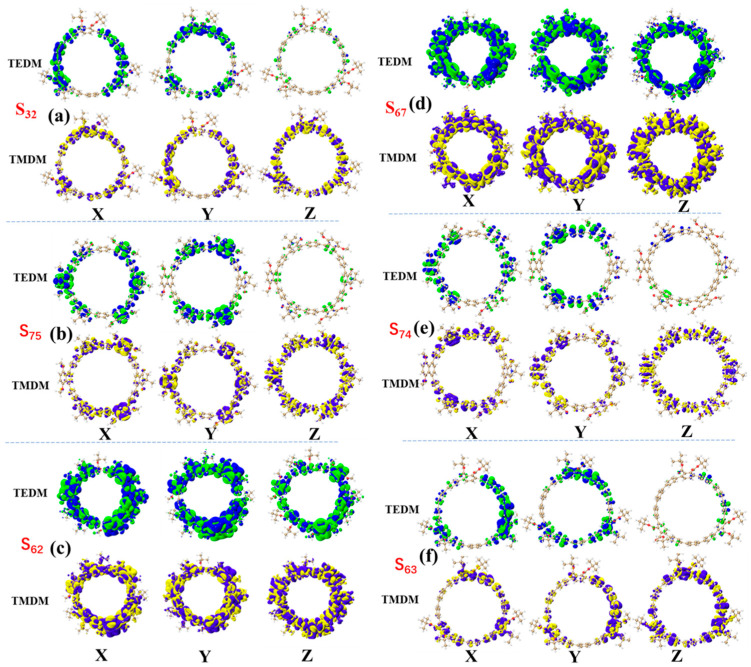
Transition electric density matrix (TEDM) and transition magnetic density matrix (TMDM) plots of [12]CPP 1a at S_32_ and S_67_ (**a**,**b**), [12]CPP 2a at S_75_ and S_72_ (**c**,**d**), and [12]CPP 3a at S_62_ and S_63_ (**e**,**f**). In the TEDM plots, green represents positive values and blue represents negative values. In the TMDM plots, yellow represents positive values and purple represents negative values. The isosurface value is 0.05.

Figure 9 presents the density distribution maps of transition electric dipole moments (TEDM) and transition magnetic dipole moments (TMDM) for [12]CPP 1a, 2a, and 3a in various excited states. By decomposing the electromagnetic interactions during the excited-state transitions, this figure provides an in-depth understanding of the mechanisms underlying chiral electromagnetic interactions and their relationship with molecular structures. Additionally, by correlating the positive and negative peak information from the electronic circular dichroism (ECD) spectra, the correspondence between chiral characteristics and the distribution of TEDM/TMDM is further analyzed.

For [12]CPP 1a, the S_32_ excited state corresponds to the positive peak in the ECD spectrum. From the TEDM and TMDM maps, it can be observed that the TEDM distribution is primarily concentrated on the left and right sides (X-direction) and the upper and lower sides (Y-direction) of the [12]CPP ring, while the Z-direction component is relatively weak. The TMDM distribution exhibits significant complementarity to the TEDM, with notable features in both the X and Y directions. In the S_67_ excited state (corresponding to the negative peak in the ECD spectrum), the TEDM and TMDM distributions are more dispersed compared to the S_32_ excited state, but their complementary relationship remains pronounced. For [12]CPP 2a, its S_75_ excited state corresponds to the positive peak in the ECD spectrum, while the S_72_ excited state corresponds to the negative peak. From the TEDM and TMDM maps, it is evident that the TEDM/TMDM distribution characteristics of [12]CPP 2a are generally consistent with those of [12]CPP 1a. However, due to the introduction of the imide acceptor, there are also distinct positive and negative isosurface distributions on the acceptor and donor, indicating that the polarization effect between the donor and acceptor modulates the electromagnetic interactions. For [12]CPP 3a, its S_62_ excited state corresponds to the positive peak in the ECD spectrum. From the TEDM and TMDM distributions, it can be seen that the TEDM is mainly concentrated around the [12]CPP host ring, exhibiting high symmetry. The TMDM distribution overlaps significantly with the positive and negative regions of the TEDM, and its intensity is relatively strong. In the S_63_ excited state (corresponding to the negative peak in the ECD spectrum), the negative isosurfaces of the TMDM increase significantly, directly leading to the negative peak in the ECD spectrum.

The absolute value of the molecular tensor product represents the intensity of photoexcitation; the larger the absolute value of the tensor product, the stronger the excitation intensity. The sign of the tensor product (positive or negative) indicates the chirality (positive or negative). Table 9 presents the TEDM/TMDM values and the characteristic values of the total tensor product for the highest excited states corresponding to the positive and negative peaks of [12]CPP 1a, [12]CPP 2a, and [12]CPP 3a. From the table, it can be observed that the absolute value of the tensor product is highest for [12]CPP 3a and lowest for [12]CPP 1a, which is entirely consistent with the intensity and direction of the ECD spectra.

The intrinsic characteristics of the transitions at the positive absorption peaks in the ECD spectra (Table 10) were analyzed by quantifying the TEDM and TMDM contributions from individual donor and acceptor fragments. In the S_32_ excited state of 1a, the Z-component of the transition electric dipole moment is dominated by a negative value from the donor, while the acceptor contributes a smaller positive value in the Z-direction; the positive TMDM value arises primarily from the Z-direction contributions of the acceptor and [12]CPP. Notably, the magnitude of the transition magnetic dipole moment in 1a is significantly larger than that of the electric dipole moment, resulting in 1a exhibiting positive chirality in the S32 state. For the 2a structure in the S_75_ excited state, the transition dipole moment also shows dominant contribution along the positive *Z*-axis: its negative electric dipole moment stems from the donor’s Z-component, and the positive magnetic dipole moment similarly originates from the combined Z-direction contributions of the acceptor and [12]CPP—with the acceptor’s contribution being more prominent in 2a—indicating that its chirality is primarily governed by the positive direction of the magnetic dipole moment. In the S_62_ excited state of 3a, the transition dipole moment is dominated by the positive *Z*-axis component: although [12]CPP contributes to both positive and negative Z-directions, the positive contribution is more pronounced, leading to positive chirality in the S_62_ state. This characteristic correlation between electric and magnetic dipole moment orientations highlights both the commonalities and differences in transition mechanisms among the structures.

The analysis of the contributions of donor/acceptor fragments to the TEDM and TMDM in the negative absorption peaks of the ECD spectra, as shown in Table S1, reveals the following: In the S_67_ excited state of 1a, the TEDM and TMDM contributions from [12]CPP and the acceptor in the Z direction jointly constitute the major contributions. Notably, the acceptor exhibits the largest negative contribution to the TMDM in the Z-direction. Similarly, the characteristics of the negative absorption peaks of 2a exhibit a highly consistent contribution pattern with those of 1a. That is, there is a synergistic effect of the electromagnetic dipole moments of the [12]CPP and acceptor fragments in the *Z*-axis direction, and the contribution of the TMDM is significantly higher than that of the TEDM. In contrast, the negative absorption mechanism of 3a in the S_63_ state differs significantly. The main driving force is the negative contribution of the TMDM of [12]CPP along the Z axis, while the contribution degree of the electromagnetic dipole moment of the acceptor is relatively low. These fragment contribution differences that are dependent on the molecular structure reveal the composition characteristics of the transition dipole moments in the negative absorption peaks. For 1a and 2a, the electromagnetic dipole moments are characterized by the synergistic contribution among fragments, and the magnetic dipole moment plays a dominant role. In contrast, 3a shows the dominant role of the [12]CPP fragment in the dimension of TMDM, reflecting the differential influence mechanism of functional fragments in different molecular structures on the characteristics of negative absorption.

## 3. Materials and Methods

To gain a comprehensive understanding of the electronic structures and photophysical properties of [12]CPP derivatives, systematic computational analyses were conducted using density functional theory (DFT) [26] and time-dependent DFT (TD-DFT) [27]. All calculations were performed with the Gaussian 16 software (Gaussian Inc., Wallingford, CT, USA) package [28]. An implicit solvent model (SMD) with chloroform as the solvent was employed during both geometry optimization and excited-state calculations to better approximate experimental conditions [12]. Molecular geometries were optimized at the B3LYP functional [29] level with the 6-311G (d, p) basis set [30], incorporating Grimme’s DFT-D3 dispersion correction [31] to account for van der Waals interactions. Multiwfn 3.8(dev) software (Beijing Kein Research Center for Natural Sciences, Beijing, China) [32] was employed to calculate HOMO–LUMO energy levels [33] and density of states (DOS) distributions. For excited-state calculations, the CAM-B3LYP functional [34] and the same 6-311G (d, p) basis set were utilized, yielding results consistent with experimental observations.

In one-photon absorption (OPA) and two-photon absorption (TPA) analyses, the transition density matrix (TDM) method [35] and electron–hole density maps [36] were applied to visualize the spatial distribution of electrons and holes across different excited states. To elucidate intermolecular charge transfer mechanisms, the intrinsic fragment charge transfer (IFCT) method [37] was adopted to quantify electron transfer between donor, acceptor, and [12]CPP fragments, as well as intra-fragment electronic redistribution under excitation. For chiral optical properties, contributions from transition electric dipole moments (TEDM) and transition magnetic dipole moments (TMDM) were decomposed using TEDM/TMDM analysis. The correlation between chiral optical signals and molecular structures was further interpreted through the positive/negative peak distributions in electronic circular dichroism (ECD) spectra [38].

All one-dimensional (1D) and two-dimensional (2D) plots were generated using Origin 2023 (OriginLab Corporation, Northampton, MA, USA), while three-dimensional (3D) visualizations were rendered with Visual Molecular Dynamics (VMD) version 1.9.4 (University of Illinois at Urbana-Champaign, Urbana, IL, USA) [39]. Notably, solvent effects and temperature-dependent influences on excited states were not considered in this study.

## 4. Conclusions

This study systematically investigated the molecular structures, photophysical properties, and chiroptical characteristics of three [12]CPP derivatives ([12]CPP 1a, [12]CPP 2a, and [12]CPP 3a), and reveals the significant influence of donor–acceptor (D–A) structural design on molecular performance through theoretical calculations. The research demonstrates that the introduction of donor–acceptor structures significantly reduces the HOMO–LUMO energy gap of the molecules, enhancing their charge transfer capabilities and optical response intensity. Among them, [12]CPP 2a, due to the strong electron-withdrawing ability of the imide acceptor, exhibits a notable redshift in both one-photon and two-photon absorption spectra, a decrease in absorption intensity, and a pronounced electron–hole separation effect, showcasing excellent charge transfer characteristics. In contrast, the donor–acceptor polarization effect in [12]CPP 1a results in a certain degree of redshift in the absorption spectra and enhanced chiroptical signal intensity, particularly demonstrating strong chiral activity in the lower wavelength region. In contrast, [12]CPP 3a, due to the absence of a donor–acceptor alternating design, exhibits photophysical properties dominated by localized excitation, with chiral signals being prominent in the higher wavelength region and the absorption intensity being the strongest among the three. TEDM/TMDM analysis further elucidates the electromagnetic interaction mechanisms in the excited states of the molecules, demonstrating that the donor–acceptor structure not only significantly regulates the intensity and distribution of chiral signals but also influences the polarization characteristics of the molecules during optical transitions. Alternating D–A CPPs hold potential applications in optoelectronic devices (such as polarization-sensitive detectors) and chiral catalysis, and their tunable two-photon absorption properties provide a new material platform for bioimaging. This study provides theoretical support for the optical and chiroptical modulation of functionalized CPP materials. Future work may incorporate experimental validation and dynamic simulations to gain deeper insight into excitation processes, and further expand their application potential in fields such as bioimaging, polarization detection, and chiral catalysis through external regulation and structural design.

## Figures and Tables

**Table 1:** Inner diameters (Å) in [12]CPP 1a, [12]CPP 2a and [13]CPP 3a.

Distance	[12]CPP 1a	[12]CPP 2a	[13]CPP 3a	
*d*_A_	16.3097	16.5051	16.4060	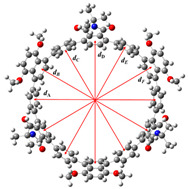
*d*_B_	16.3613	16.3304	16.3220	
*d*_C_	16.7210	16.5163	16.3541	
*d*_D_	16.4695	16.3256	16.3541	
*d*_E_	16.3635	16.4972	16.4953	
*d*_F_	15.9345	16.3096	16.4300	
D.P.	1.0494	1.0127	1.0106	

**Table 2:** Transition indices of three molecules in S_2_ and S_3_ states for one-photon absorption.

		*D* (A)	*S_r_*	*H* (A)	*t* (A)	*E_coul_* (eV)	*HDI*	*EDI*
[12]CPP 1a	S_2_	0.368	0.785	8.386	−3.624	1.947	3.37	3.24
	S_3_	0.453	0.782	8.124	−3.097	2.068	3.55	3.37
[12]CPP 2a	S_2_	0.979	0.811	8.087	−1.497	2.063	4.57	4.34
	S_3_	1.080	0.808	6.737	−1.778	2.374	5.34	5.18
[12]CPP 3a	S_2_	0.251	0.834	8.318	−4.054	2.067	3.52	3.29
	S_3_	0.188	0.842	7.865	−6.111	2.215	3.85	3.63

**Table 3:** Net electron transfer amounts between different fragments in the S_2_ excited state (Unit: eV).

	[12]CPP 1a	[12]CPP 2a	[12]CPP 3a
Donor→Acceptor	0.024	0.157	——
Donor→[12]CPP	0.089	0.026	——
Acceptor→[12]CPP	−0.003	−0.061	−0.095

——: It indicates no value.

**Table 4:** Net changes in the number of electrons of fragments in the S_2_ excited state (Unit: eV).

	[12]CPP 1a	[12]CPP 2a	[12]CPP 3a
Donor	−0.114	−0.183	——
Acceptor	0.027	0.218	0.095
[12]CPP	0.087	−0.035	−0.095

**Table 5:** Electron redistribution amounts within fragments in the S_2_ excited state (Unit: eV).

	[12]CPP 1a	[12]CPP 2a	[12]CPP 3a
Donor	0.560	0.267	——
Acceptor	0.001	0.043	0.046
[12]CPP	0.043	0.058	0.606

**Table 6:** Percentages of charge transfer and local excitation in the system in the S_2_ excited state.

	[12]CPP 1a	[12]CPP 2a	[12]CPP 3a
Charge transfer	39.018%	63.247%	9.520%
Local excitation	60.814%	36.753%	90.480%

**Table 7:** NPA charge distribution of the system.

	[12]CPP 1a	[12]CPP 2a	[12]CPP 3a
Donor	0.082	0.118	——
Acceptor	−0.185	−0.179	−0.099
[12]CPP	0.103	0.061	0.099

**Table 8:** The main two-photon absorption process and its transition dipole moment value.

Molecule	TPA States	Process	Integral Value
1a	S_15_	$\langle \phi_{S_{0}}\left|\mu \right|\phi_{S_{2}}\rangle \times \langle \phi_{S_{2}}\left|\mu \right|\phi_{S_{15}}\rangle$	24.26 × 2.80
2a	S_22_	$\langle \phi_{S_{0}}\left|\mu \right|\phi_{S_{2}}\rangle \times \langle \phi_{S_{2}}\left|\mu \right|\phi_{S_{22}}\rangle$	14.51 × 5.84
3a	S_25_	$\langle \phi_{S_{0}}\left|\mu \right|\phi_{S_{2}}\rangle \times \langle \phi_{S_{2}}\left|\mu \right|\phi_{S_{25}}\rangle$	30.78 × 16.84

**Table 9:** TEDM/TMDM values and tensor product eigenvalues of [12]CPP 1a, [12]CPP 2a, and [12]CPP 3a under different excited states.

			X	Y	Z	Eigenvalue
[12]CPP 1a	S_32_	TEDM	0.1632	0.2092	−0.8479	3.91512528
		TMDM	1.2061	−0.7311	4.6692	
	S_67_	TEDM	−0.2380	0.8016	−1.2801	−4.47624147
		TMDM	−1.1388	−0.9480	−3.8787	
[12]CPP 2a	S_75_	TEDM	−0.1188	0.0578	−1.1666	1.41938294
		TMDM	−0.1259	−0.8377	1.1880	
	S_74_	TEDM	0.2136	−0.0725	−0.5302	−1.61735229
		TMDM	0.1990	1.3011	−3.1482	
[12]CPP 3a	S_62_	TEDM	0.5917	−0.3651	−1.7692	6.76021598
		TMDM	0.7606	2.0044	3.6618	
	S_63_	TEDM	−0.4403	0.7902	−0.9817	−6.92439249
		TMDM	−3.0439	−1.3106	−6.7432	

**Table 10:** The contributions of transition dipole moments from different donor–acceptor [12]CPP fragments in the structures of [12]CPP 1a, [12]CPP 2a, and [12]CPP 3a.

			Total	[12]CPP	Donor	Acceptor
1a	TEDM	X	0.175	0.006	0.289	−0.108
		Y	0.226	−0.232	0.563	−0.123
		Z	−0.907	−0.104	−1.033	0.223
	TMDM	X	1.204	−0.189	1.914	−0.519
		Y	−0.733	0.0722	−1.351	0.548
		Z	4.662	1.810	0.665	2.187
2a	TEDM	X	−0.119	0.0880	−0.061	−0.146
		Y	0.0642	−0.002	0.015	0.051
		Z	−1.213	−0.258	−0.750	−0.205
	TMDM	X	−0.127	−0.009	−0.072	−0.046
		Y	−0.833	−0.206	−0.259	−0.367
		Z	4.1725	1.300	0.214	2.660
3a	TEDM	X	0.600	0.241	——	0.270
		Y	−0.356	−0.443	——	0.096
		Z	−1.842	−1.034	——	−0.775
	TMDM	X	0.749	1.102	——	−0.354
		Y	1.986	0.970	——	1.034
		Z	3.675	2.600	——	1.070

## Data Availability

The original contributions presented in this study are included in the article/Supplementary Material. Further inquiries can be directed to the corresponding author(s).

## Supplementary Materials

The following supporting information can be downloaded at: https://www.mdpi.com/article/10.3390/molecules30153127/s1, Figure S1. The ChemDraw structural diagram of the donor-acceptor (D–A) nanoring, in which the strong donor groups are highlighted in blue and the strong acceptor groups are highlighted in red. Figure S2. Schematic diagrams of [12]CPP 1a (a) and [12]CPP 3a (b) defining the inner diameter. Figure S3. The dihedral angles of [12]CPP 1a, [12]CPP 2a and [12]CPP 3a relative to the benzene ring. Figure S4. Energy diagrams for the molecular orbitals (MOs) of [12]CPP 1a, 2a, and 3a. Figure S5. The comparison between the calculated one-photon absorption spectra (a,b) and experimental data (c,d) for [12]CPP 1a and [12]CPP 2a. Figure S6. Jablonski Diagram of Two-Photon Absorption. Figure S7. Schematic diagrams of the structures of the enantiomers of [12]CPP 1a, [12]CPP 2a and [12]CPP 3a. Figure S8. The ECD spectra of [12]CPP 1a, [12]CPP 2a, [12]CPP 3a and their enantiomers. Table S1. In the structures of [12]CPP 1a, [12]CPP 2a, and [12]CPP 3a, the contributions of different donor-acceptor and [12]CPP fragments to the transition dipole moments of the excited states corresponding to the negative absorption peaks. Table S2. Relaxed structures of [12]CPP 1a. Table S3. Relaxed structures of [12]CPP 2a. Table S4. Relaxed structures of [12]CPP 3a.
