# Supplementary material for: Precision Molecular Engineering of Alternating Donor–Acceptor Cycloparaphenylenes: Multidimensional Optoelectronic Response and Chirality Modulation via Polarization-Driven Charge Transfer
Source: Molecules. 2025 Jul 25;30(15):3127. doi: 10.3390/molecules30153127 (PMC12348527; doi:10.3390/molecules30153127)
Supplement: Supplementary file 1 [file molecules-30-03127-s001.zip › molecules-3677870-supplementary.pdf]

Supporting Information

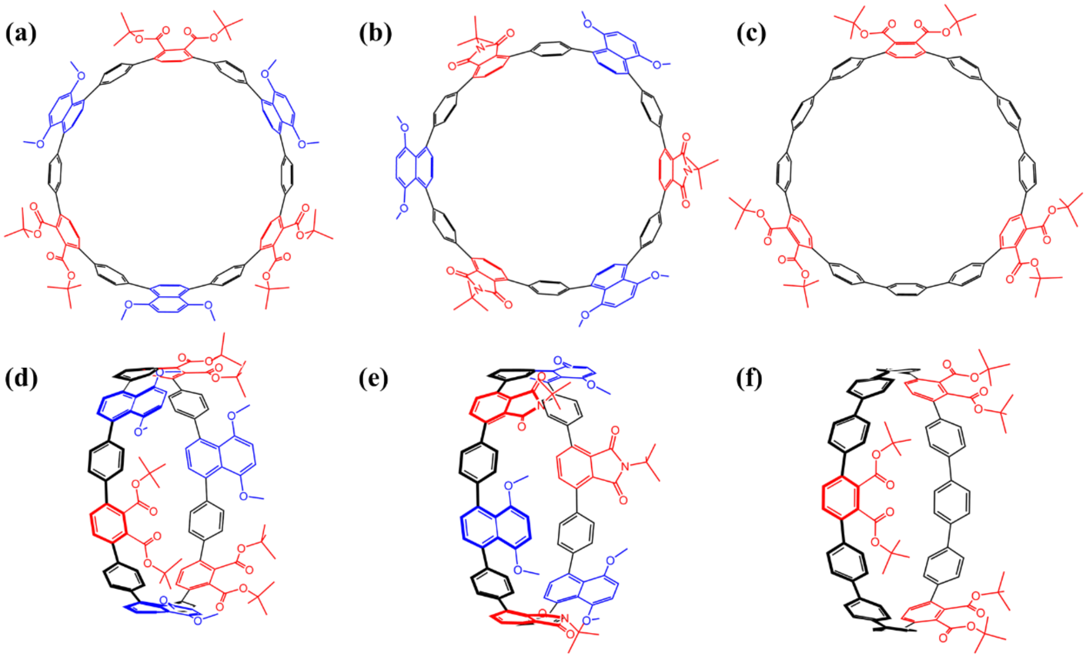

Figure S1. The ChemDraw structural diagram of the donor-acceptor (D-A) nanoring, in which the strong donor groups are highlighted in blue and the strong acceptor groups are highlighted in red.

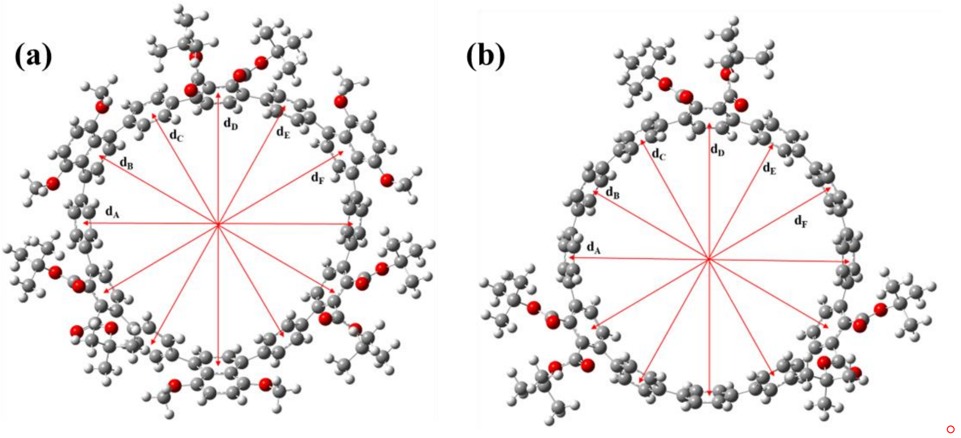

Figure S2. Schematic diagrams of [12]CPP 1a (a) and [12]CPP 3a (b) defining the inner diameter.

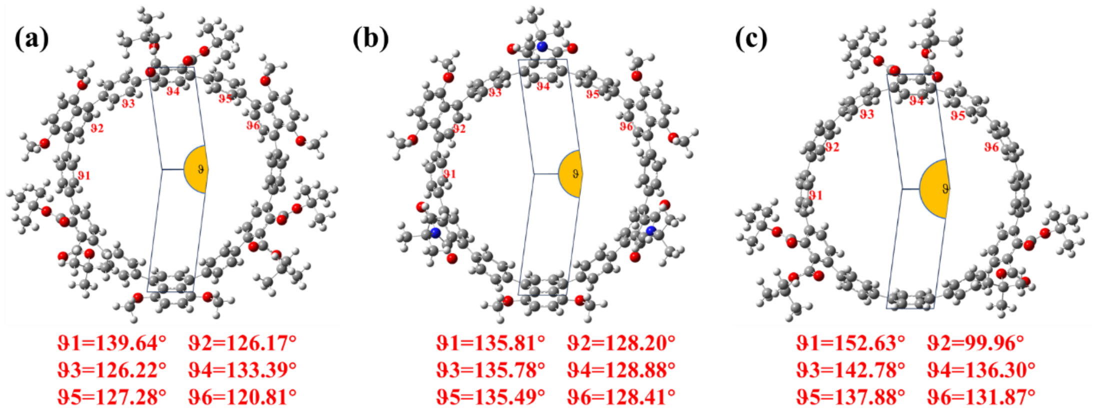

Figure S3. The dihedral angles of [12]CPP 1a, [12]CPP 2a and [12]CPP 3a relative to the benzene ring.

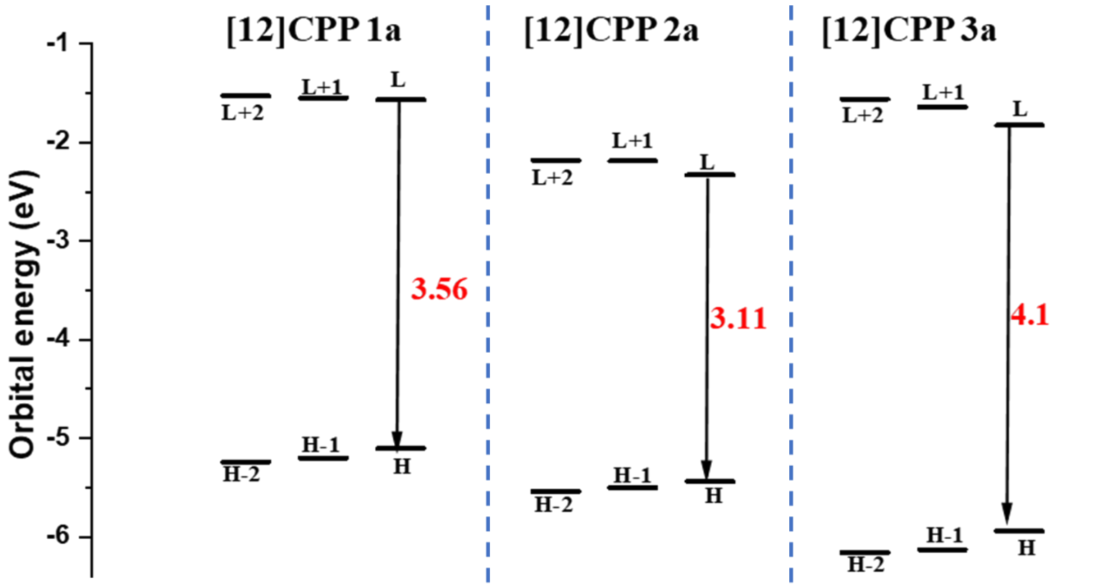

Figure S4. Energy diagrams for the molecular orbitals (MOs) of [12]CPP 1a, 2a, and 3a. Arrows represent the energy difference between the highest occupied molecular orbital (HOMO) and the lowest unoccupied molecular orbital (LUMO). H denotes HOMO, and L denotes LUMO.

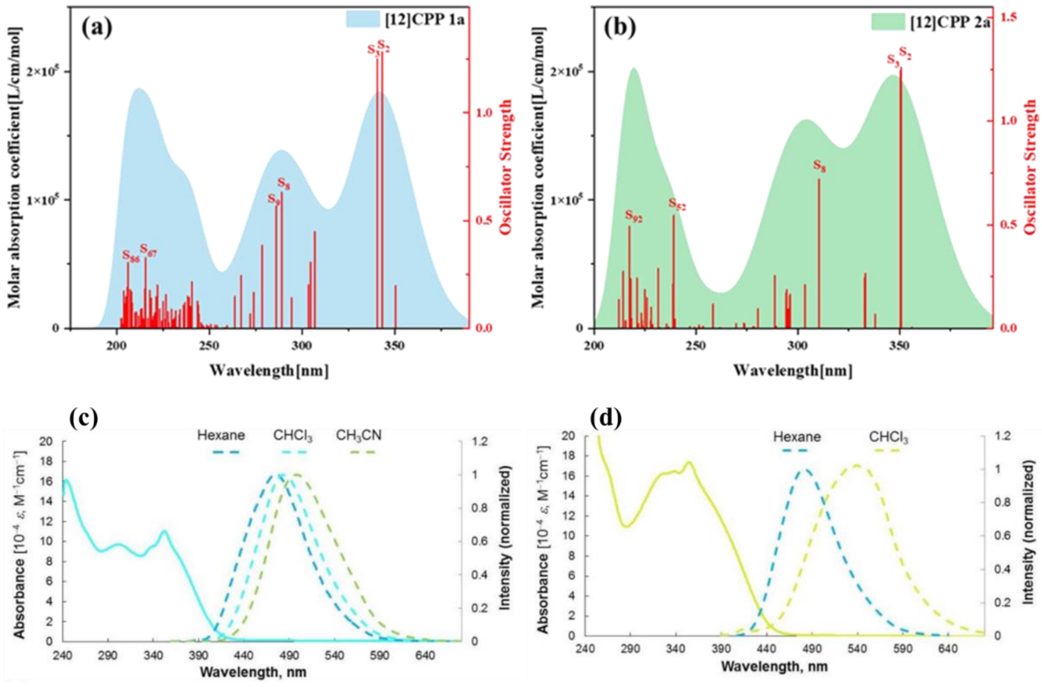

Figure S5. The comparison between the calculated one-photon absorption spectra (a, b) and experimental data (c, d) for [12]CPP 1a and [12]CPP 2a. In panels (c) and (d), the solid lines represent the experimental absorption spectra of [12]CPP 1a and [12]CPP 2a in chloroform, while the dashed lines correspond to their fluorescence spectra measured in different solvents.

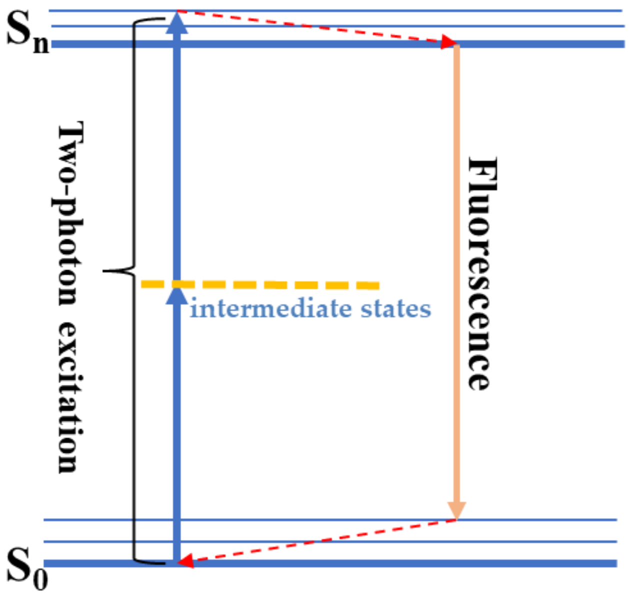

Figure S6. Jablonski Diagram of Two-Photon Absorption.

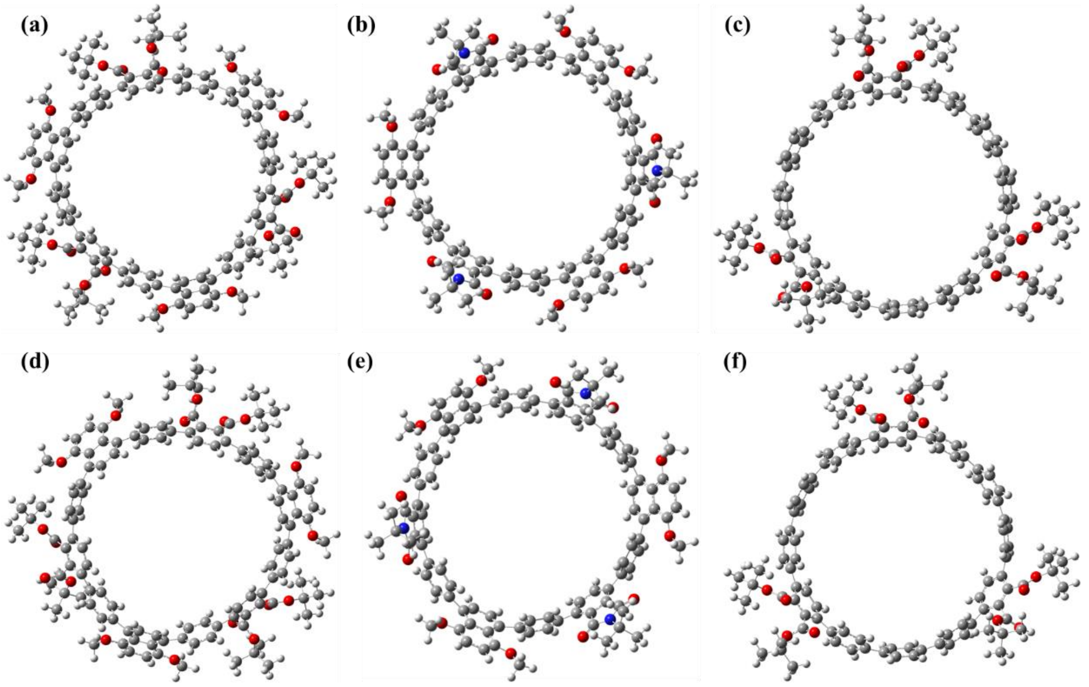

Figure S7. Schematic diagrams of the structures of the enantiomers of [12]CPP 1a, [12]CPP 2a and [12]CPP 3a.

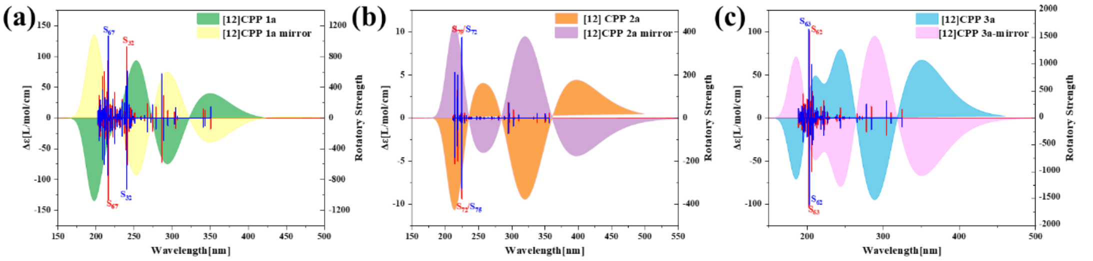

Figure S8. The ECD spectra of [12]CPP 1a, [12]CPP 2a, [12]CPP 3a and their enantiomers.

Table S1. In the structures of [12]CPP 1a, [12]CPP 2a, and [12]CPP 3a, the contributions of different donor-acceptor and [12]CPP fragments to the transition dipole moments of the excited states corresponding to the negative absorption peaks.

			Total	12CPP	Donor	Acceptor
1a	TEDM	X	-0.231	-0.611	0.168	0.197
		Y	0.814	0.305	0.033	0.497
		Z	-1.365	-0.510	-0.002	-0.845
	TMDM	X	-1.104	-0.565	-0.306	-0.233
		Y	-0.931	-0.168	-0.161	-0.603
		Z	-3.878	-1.252	-0.914	-1.708
2a	TEDM	X	0.217	-0.132	0.070	0.279
		Y	-0.077	-0.004	-0.009	-0.064

		Z	-0.566	-0.148	-0.324	-0.093
		X	0.193	0.025	0.092	0.075
	TMDM	Y	1.303	0.358	0.348	0.597
		Z	-3.095	-0.757	-0.599	-1.740
		X	-0.429	-0.011	--	-0.351
	TEDM	Y	0.787	0.031	--	0.753
3a		Z	-1.044	-0.533	--	-0.540
		X	-3.008	-0.590	--	-2.415
	TMDM	Y	-1.306	-0.495	--	-0.809
		Z	-6.740	-4.751	--	-1.983

Table S2. Relaxed structures of [12]CPP 1a.

C	0.876377	-8.50132	1.414718
C	1.704667	-8.93857	0.325563
C	3.105167	-8.60151	0.323377
C	3.646392	-7.85441	1.423989
C	2.864837	-7.69581	2.557543
C	1.496639	-8.01016	2.551959
C	-0.76837	-10.6871	-1.75499
C	1.178619	-9.71726	-0.75816
C	1.979946	-10.0608	-1.82531
C	3.348882	-9.72221	-1.83402
C	3.916097	-9.04313	-0.77616
C	6.106131	-9.26555	-1.7086
C	4.850192	-6.98503	1.335935
C	4.922143	-6.0673	0.27392
C	5.82697	-5.01813	0.295139
C	6.712833	-4.84923	1.373665
C	6.691156	-5.80154	2.400784
C	5.774243	-6.856	2.381001
C	7.442114	-3.55691	1.498384
C	8.307595	-3.02411	0.523439
C	8.70798	-1.67582	0.580183
C	8.248837	-0.83281	1.610969
C	7.518775	-1.41666	2.656656
C	7.126306	-2.74603	2.600537
C	8.97758	-3.9211	-0.48744
C	8.546068	-5.43112	-2.37666
C	7.23341	-5.76884	-3.08014
C	9.11294	-6.66339	-1.67196
C	9.53967	-4.78629	-3.34095
C	9.570654	-1.15555	-0.53181
C	11.44968	0.373256	-0.96849
C	12.34376	-0.64639	-1.67175
C	12.24918	1.234089	0.006648
C	10.66981	1.250037	-1.9482
C	8.300019	0.648774	1.544646
C	7.838717	1.29397	0.384476
C	7.608653	2.661605	0.371986
C	7.824568	3.443134	1.520632
C	8.371219	2.813564	2.648181
C	8.600751	1.437525	2.66253
C	7.216084	4.797823	1.588379
C	7.22734	5.750969	0.513709
C	6.180139	6.738266	0.437606
C	5.163229	6.764989	1.450882
C	5.352948	5.999286	2.590114
C	6.360464	5.025704	2.654923
C	10.39338	4.94377	-1.18593
C	8.264336	5.769542	-0.4784
C	8.206338	6.656541	-1.53251
C	7.171902	7.611641	-1.61642
C	6.202388	7.686143	-0.63959
C	5.112695	9.528036	-1.71037
C	3.798658	7.328003	1.268684
C	3.056805	6.945816	0.138872
C	1.688174	7.161664	0.07068
C	1.003752	7.784817	1.129441
C	1.757168	8.243621	2.220707
C	3.131952	8.018426	2.289823
C	-0.48157	7.742329	1.190846
C	-1.34437	8.121863	0.142793
C	-2.72733	7.875666	0.222391
C	-3.26719	7.174741	1.32345
C	-2.41851	6.899416	2.402863
C	-1.06373	7.191288	2.343363

C	-0.7769	8.849938	-1.05185
C	-0.48699	11.13708	-1.90747
C	1.041485	11.08663	-1.91393
C	-0.98906	12.46525	-1.3472
C	-1.09646	10.86008	-3.28033
C	-3.60132	8.379287	-0.88632
C	-5.87667	9.234137	-1.30048
C	-5.46461	10.51549	-2.02363
C	-7.00749	9.498199	-0.30877
C	-6.25316	8.109587	-2.26475
C	-4.59905	6.522129	1.308534
C	-4.95916	5.733629	0.201727
C	-6.05669	4.887602	0.254849
C	-6.83742	4.787204	1.419764
C	-6.53193	5.639809	2.489946
C	-5.42846	6.491754	2.437102
C	-7.74171	3.617809	1.576702
C	-8.62998	3.124505	0.561593
C	-9.02527	1.737859	0.583898
C	-8.52036	0.880355	1.619826
C	-7.88377	1.46737	2.702144
C	-7.50063	2.815722	2.681125
C	-9.41611	6.200978	-1.30606
C	-9.16789	3.979009	-0.45855
C	-9.99069	3.464294	-1.43767
C	-10.3777	2.10851	-1.41541
C	-9.94222	1.266384	-0.41455
C	-11.333	-0.51307	-1.1995
C	-8.36533	-0.59326	1.501634
C	-7.77401	-1.11963	0.339735
C	-7.28037	-2.41512	0.313857
C	-7.36311	-3.24413	1.446368
C	-8.03301	-2.75349	2.574498
C	-8.52928	-1.44976	2.599652
C	-6.56544	-4.49419	1.47832
C	-6.49126	-5.39876	0.395926
C	-5.43531	-6.3241	0.312262
C	-4.47503	-6.41865	1.339742
C	-4.66452	-5.62457	2.481412
C	-5.67398	-4.67441	2.543288
C	-7.52448	-5.40414	-0.68968
C	-9.92151	-4.96686	-1.06366
C	-9.68087	-3.83686	-2.06439
C	-11.0041	-4.57865	-0.05906
C	-10.2487	-6.2951	-1.74428
C	-5.35439	-7.27059	-0.86156
C	-6.30584	-9.40381	-1.63219
C	-6.73932	-8.88914	-3.00318
C	-7.39335	-10.2749	-1.00902
C	-4.96651	-10.1411	-1.66993
C	-3.22071	-7.21233	1.252764
C	-2.77469	-7.99345	2.329406
C	-1.47919	-8.51177	2.352887
C	-0.58689	-8.26298	1.301181
C	-1.06166	-7.54243	0.192187
C	-2.34685	-7.02345	0.167456
O	-0.11611	-10.1224	-0.62705
O	5.252926	-8.78965	-0.67748
O	8.122906	-4.43087	-1.3708
O	10.17283	-4.14485	-0.4278
O	10.51874	-0.3401	-0.06692
O	9.404063	-1.46824	-1.6991
O	9.303571	4.908431	-0.27585
O	5.253045	8.662749	-0.59341
O	-0.9989	10.16179	-0.91781
O	-0.16382	8.31273	-1.9517
O	-4.77935	8.79579	-0.41279
O	-3.24721	8.428911	-2.05253
O	-8.85845	5.303985	-0.35653
O	-10.3787	-0.01845	-0.27086
O	-8.73722	-5.13788	-0.19619
O	-7.27311	-5.65931	-1.85572
O	-6.21323	-8.27864	-0.67434
O	-4.57984	-7.14844	-1.78848
H	7.115683	-8.9753	-1.41474
H	5.864286	-8.8092	-2.67648
H	8.978894	6.664899	-2.29142
H	4.207309	-6.14022	-0.53968
H	7.185493	8.318806	-2.43666
H	5.815061	-4.28463	-0.50125
H	7.37457	-5.70048	3.239656
H	4.601682	6.023822	3.373443
H	5.753504	-7.56401	3.204928
H	6.349881	4.323484	3.482636
H	10.08437	4.655537	-2.19853
H	11.11782	4.220654	-0.80841
H	10.85496	5.939251	-1.21688
H	7.167046	-0.78781	3.468646

H	4.927745	8.963359	-2.63285
H	5.998397	10.163	-1.843
H	4.246724	10.1558	-1.49052
H	1.583308	-10.6395	-2.65064
H	3.954541	-10.0469	-2.67084
H	6.483566	-3.14887	3.377332
H	3.551172	6.406085	-0.6628
H	7.587856	0.704525	-0.49256
H	1.13042	6.794892	-0.78129
H	3.269407	-7.14811	3.403271
H	7.182534	3.122765	-0.51303
H	0.886212	-7.69153	3.391475
H	1.256701	8.752457	3.040085
H	-1.81092	-10.819	-1.45847
H	-0.34124	-11.6623	-2.02269
H	-0.71832	-10.0169	-2.62234
H	8.582056	3.39972	3.538276
H	8.991322	0.967829	3.561038
H	3.687519	8.351999	3.161933
H	6.051846	-10.3582	-1.79904
H	-12.2493	0.091142	-1.19107
H	-11.5659	-1.5273	-0.87216
H	-10.9269	-0.5428	-2.21847
H	-2.81271	6.36359	3.260625
H	-0.41932	6.904542	3.168319
H	-7.6312	-0.47677	-0.5228
H	-6.75564	-2.76593	-0.57004
H	-8.142	-3.38593	3.451184
H	-9.01385	-1.07932	3.4987
H	-3.94562	-5.69442	3.291742
H	-5.71642	-3.99327	3.387474
H	-2.66452	-6.42037	-0.67332
H	-0.38659	-7.32938	-0.63059
H	-3.43862	-8.17409	3.170509
H	-1.14896	-9.09084	3.210876
H	-4.32698	5.729788	-0.68144
H	-6.27004	4.236167	-0.58652
H	-5.19257	7.117541	3.293342
H	-7.1399	5.607163	3.38977
H	-10.3914	4.102004	-2.21579
H	-11.0617	1.75494	-2.17664
H	-7.51211	0.835204	3.502427
H	-6.84636	3.181291	3.466342
H	-10.5132	6.172118	-1.28667
H	-9.06541	5.982426	-2.32252
H	-9.07546	7.194221	-1.01025
H	-11.9554	-4.41968	-0.57739
H	-11.144	-5.37072	0.684108
H	-10.7296	-3.65779	0.465199
H	-8.89842	-4.09542	-2.77993
H	-10.6068	-3.64588	-2.61791
H	-9.39636	-2.91935	-1.54117
H	-9.48842	-6.56006	-2.48098
H	-10.3171	-7.09692	-1.00092
H	-11.2162	-6.21603	-2.25246
H	-7.10096	-10.6014	-0.00553
H	-8.33453	-9.72003	-0.93333
H	-7.56223	-11.1622	-1.6278
H	-5.96991	-8.26334	-3.45702
H	-6.93424	-9.74257	-3.66228
H	-7.65902	-8.303	-2.91549
H	-4.18723	-9.52513	-2.1215
H	-4.65605	-10.4133	-0.6552
H	-5.07311	-11.0606	-2.25589
H	9.113699	-3.87451	-3.77169
H	10.47271	-4.53132	-2.83566
H	9.759713	-5.48421	-4.15652
H	9.265158	-7.4605	-2.4081
H	10.06813	-6.44764	-1.19104
H	8.408611	-7.0224	-0.91452
H	6.518177	-6.20227	-2.37475
H	6.789087	-4.87015	-3.52061
H	7.417475	-6.49171	-3.88172
H	12.99484	1.822008	-0.53869
H	11.58719	1.918718	0.54676
H	12.77021	0.60769	0.738196
H	11.37596	1.870131	-2.51138
H	10.09678	0.649146	-2.65599
H	9.986247	1.910818	-1.40725
H	12.8292	-1.29708	-0.93663
H	11.76942	-1.26638	-2.36245
H	13.12358	-0.12165	-2.23487
H	-0.8215	11.67006	-3.96522
H	-0.74102	9.915098	-3.69274
H	-2.18766	10.8216	-3.21058
H	-0.59168	12.6367	-0.34135
H	-0.66784	13.28898	-1.99293
H	-2.08296	12.47176	-1.29426

H	1.428309	11.20786	-0.89617	H	-6.49157	7.196884	-1.71097
H	1.405665	10.14014	-2.31675	H	-7.13928	8.408868	-2.83522
H	1.430313	11.90472	-2.53029	H	-5.44577	7.898434	-2.96803
H	-5.10853	11.26072	-1.3039	H	-6.71982	10.27472	0.407823
H	-4.67542	10.32264	-2.75231	H	-7.90186	9.835531	-0.84279
H	-6.3313	10.93304	-2.54803	H	-7.25087	8.587659	0.247839

Table S3. Relaxed structures of [12]CPP 2a.

C	8.089114	-7.44892	1.520741	C	-7.19861	-4.67997	-2.42154
C	1.904409	-10.8336	1.517459	C	-8.41735	-1.21343	-1.50606
C	5.950809	-7.95056	0.567639	C	-9.11224	-0.49415	-0.47783
C	5.628726	-8.8286	1.57658	C	-9.07356	0.943952	-0.47432
C	4.392156	-9.50423	1.57691	C	-8.34276	1.629822	-1.50039
C	3.479298	-9.30061	0.568028	C	-7.90127	0.898396	-2.58799
C	3.734199	-8.33051	-0.45607	C	-7.93745	-0.50177	-2.59056
C	2.77742	-8.04585	-1.48611	C	-10.5255	-3.2678	1.477481
C	3.192159	-7.30055	-2.57476	C	-9.86589	-1.16551	0.540182
C	4.421558	-6.62941	-2.57549	C	-10.4689	-0.44797	1.547298
C	5.274012	-6.683	-1.48762	C	-10.4301	0.960598	1.551314
C	4.996818	-7.64098	-0.45655	C	-9.78864	1.649663	0.548156
C	1.311176	-8.25421	-1.36029	C	-10.3312	3.777896	1.500095
C	0.654847	-7.7851	-0.21185	C	-7.78851	3.002764	-1.37134
C	-0.72243	-7.66327	-0.17545	C	-7.05663	3.334356	-0.22075
C	-1.50693	-8.00493	-1.28808	C	-6.26135	4.465315	-0.18108
C	-0.8619	-8.55459	-2.40197	C	-6.16206	5.317351	-1.29213
C	0.524699	-8.6817	-2.43504	C	-6.95822	5.03575	-2.40841
C	-2.91771	-7.56199	-1.34346	C	-7.76206	3.899002	-2.445
C	-3.83285	-7.62234	-0.28955	C	-5.07461	6.319112	-1.3419
C	-5.02385	-6.89484	-0.29468	C	-4.67094	7.133679	-0.28091
C	-5.38341	-6.05618	-1.3528	C	-3.4474	7.804959	-0.27765
C	-4.53674	-6.11758	-2.47751	C	-2.54126	7.707726	-1.33666
C	-3.35357	-6.84029	-2.47279	C	-3.01601	7.014022	-2.46794
C	-3.78979	-8.43588	0.963687	C	-4.2319	6.347806	-2.47079
C	-5.76425	-7.22761	0.955283	C	-5.38864	7.492222	0.975022
C	-5.41871	-8.70464	2.994187	C	-3.36087	8.608664	0.979891
C	-4.35416	-9.61184	3.622043	C	-4.96484	8.936563	3.022067
C	-5.68537	-7.5408	3.961551	C	-5.28199	7.77867	3.981417
C	-6.69046	-9.52915	2.749681	C	-6.19726	9.821986	2.789652
C	-6.42258	-5.00449	-1.30277	C	-3.85595	9.787237	3.652148
C	-7.22558	-2.98527	-0.22686	C	-1.11092	8.082641	-1.28458
C	-6.48696	-4.15409	-0.18791	C	-0.34154	7.702845	-0.17376
C	-7.93379	-2.61308	-1.37987	C	1.039862	7.759528	-0.21243
C	-7.94607	-3.50546	-2.4571	C	1.715672	8.197707	-1.36179

C	0.948467	8.663449	-2.43461	O	-6.49998	7.147799	1.316008
C	-0.44256	8.602598	-2.39896	O	-2.46533	9.355416	1.302738
C	3.170012	7.919343	-1.48969	O	2.813781	9.899745	0.4413
C	4.140032	8.157398	-0.4602	O	7.526023	7.023552	0.432874
C	5.367732	7.407582	-0.4617	O	9.462254	2.08142	1.288821
C	5.596911	6.436258	-1.49195	O	9.350569	-2.51679	1.302041
C	4.742573	6.423869	-2.57945	N	-4.5445	8.353658	1.701704
C	3.54742	7.154395	-2.57838	N	9.531868	-0.22584	1.685261
C	2.437446	10.74602	1.518119	N	-4.96497	-8.13223	1.680337
C	3.933138	9.137816	0.564943	H	6.34063	-9.05534	2.359028
C	4.856774	9.297693	1.571923	H	4.198681	-10.2257	2.359663
C	6.059585	8.563652	1.570067	H	2.486301	-7.08825	-3.36979
C	6.336917	7.670389	0.561217	H	4.623663	-5.92131	-3.37113
C	8.448982	7.064161	1.511672	H	8.929674	-6.81563	1.242267
C	6.508316	5.269023	-1.3658	H	8.44043	-8.47529	1.669167
C	6.429597	4.467976	-0.21611	H	7.645749	-7.07539	2.449256
C	7.01028	3.213282	-0.17811	H	2.580433	-11.6827	1.66209
C	7.69726	2.701785	-1.29012	H	0.918118	-11.2001	1.238027
C	7.85195	3.533493	-2.40519	H	1.82858	-10.2628	2.448444
C	7.270537	4.798721	-2.44017	H	1.240747	-7.44833	0.635587
C	8.017671	1.258125	-1.34263	H	-1.19211	-7.23125	0.699335
C	8.526507	0.497726	-0.28675	H	-1.44523	-8.86222	-3.26362
C	8.490879	-0.89735	-0.28937	H	1.002705	-9.08604	-3.32098
C	7.94477	-1.62995	-1.34609	H	-4.76557	-5.49958	-3.33824
C	7.575352	-0.86849	-2.47258	H	-2.69425	-6.76511	-3.32996
C	7.61021	0.517566	-2.47052	H	-5.89465	-4.37645	0.690848
C	9.208703	0.943119	0.966705	H	-7.20107	-2.31246	0.622583
C	9.146471	-1.37094	0.962266	H	-8.51783	-3.26334	-3.34667
C	10.25357	-0.33159	2.999761	H	-7.19859	-5.33732	-3.28476
C	10.51031	1.044481	3.624943	H	-11.0277	-0.95085	2.325294
C	11.60174	-1.02468	2.756942	H	-10.9603	1.489407	2.332161
C	9.37645	-1.14035	3.968244	H	-7.35979	1.403509	-3.37982
C	7.553839	-3.05559	-1.29233	H	-7.42213	-1.03098	-3.38407
C	6.846212	-3.53244	-0.17781	H	-9.98951	-3.08201	2.413682
C	6.204891	-4.75742	-0.21376	H	-10.4048	-4.312	1.194191
C	6.242039	-5.5621	-1.36306	H	-11.5895	-3.0487	1.61449
C	7.023995	-5.13011	-2.43947	H	-9.80828	3.554036	2.435328
C	7.665319	-3.89424	-2.40728	H	-11.4061	3.617952	1.634012
O	7.17079	-7.36357	0.440473	H	-10.1512	4.81592	1.225768
O	2.32612	-10.0095	0.440351	H	-7.06049	2.657385	0.625703
O	-2.93039	-9.22364	1.287197	H	-5.65388	4.653896	0.695158
O	-6.86011	-6.83394	1.293065	H	-6.93105	5.696349	-3.26873
O	-9.97185	-2.51469	0.408106	H	-8.34958	3.689089	-3.33249
O	-9.81938	3.003414	0.425205	H	-2.36507	6.912841	-3.32887

H	-4.49474	5.746929	-3.33387	H	-3.41738	-9.08539	3.803774
H	-0.82991	7.292698	0.701276	H	-4.74566	-9.9494	4.58502
H	1.61079	7.395046	0.633768	H	-6.48046	-6.8893	3.605569
H	-1.01202	8.939106	-3.25902	H	-5.97839	-7.9398	4.935578
H	1.443375	9.045746	-3.32106	H	-4.77863	-6.94543	4.098776
H	4.698646	10.02726	2.354941	H	-7.02771	-9.97216	3.690122
H	6.78271	8.756033	2.351464	H	-7.4974	-8.91556	2.352085
H	4.909441	5.706137	-3.37464	H	-6.48811	-10.3409	2.046082
H	2.831589	6.976256	-3.3728	H	-5.55272	8.182964	4.959707
H	3.153824	11.56242	1.656701	H	-6.10775	7.167651	3.623496
H	2.33911	10.18229	2.451247	H	-4.40341	7.140773	4.110394
H	1.4683	11.1582	1.242392	H	-7.03371	9.25016	2.390708
H	8.852041	8.071734	1.65775	H	-6.50964	10.27269	3.734997
H	9.256004	6.388567	1.233318	H	-5.95972	10.62892	2.091513
H	7.98867	6.715398	2.441566	H	-2.94439	9.215508	3.825339
H	5.846277	4.809351	0.631268	H	-3.60311	10.65772	3.050529
H	6.870065	2.592281	0.697782	H	-4.2268	10.1351	4.619601
H	8.409156	3.180029	-3.26654	H	9.206423	-2.15454	3.613497
H	7.38257	5.413083	-3.32725	H	9.868528	-1.19506	4.942234
H	7.1551	-1.37782	-3.33231	H	8.409127	-0.64939	4.104996
H	7.215956	1.049143	-3.32907	H	12.15349	-1.09515	3.697685
H	6.738558	-2.90463	0.697903	H	11.47128	-2.03066	2.361055
H	5.607177	-5.06944	0.634835	H	12.20529	-0.44638	2.052478
H	8.236678	-3.56832	-3.27019	H	11.16335	1.66592	3.01582
H	7.104615	-5.75009	-3.32601	H	9.587309	1.594764	3.806464
H	-4.13992	-10.4886	3.014566	H	10.99892	0.87477	4.587731

Table S4. Relaxed structures of [12]CPP 3a.

C	1.847675	-8.1718	-0.94913	C	-7.77811	-3.57283	-1.32203
C	1.132292	-8.57564	0.192419	C	-6.84517	-3.86191	-2.3271
C	-0.25545	-8.66677	0.174518	C	-6.04578	-4.99319	-2.25742
C	-0.98778	-8.35903	-0.98593	C	-8.26859	-2.17492	-1.23227
C	-0.26223	-8.10741	-2.16256	C	-8.10146	-1.47403	-0.02686
C	1.125391	-8.01556	-2.14449	C	-8.18377	-0.08738	0.010261
C	-2.43903	-8.06027	-0.94269	C	-8.44089	0.658177	-1.15428
C	-2.96089	-7.39308	0.178866	C	-8.7481	-0.05652	-2.32588
C	-4.21457	-6.79471	0.143358	C	-8.6547	-1.44514	-2.36681
C	-5.00555	-6.84764	-1.01575	C	-8.15549	2.112976	-1.16617
C	-4.53656	-7.61198	-2.09647	C	-8.22686	2.900463	-0.0026
C	-3.27946	-8.2116	-2.05918	C	-7.61575	4.148799	0.05967
C	-6.1274	-5.88207	-1.17367	C	-6.91259	4.668079	-1.04169
C	-7.16481	-5.68144	-0.24204	C	-6.97404	3.9482	-2.24668
C	-7.99421	-4.54823	-0.32594	C	-7.57927	2.698279	-2.30686

C	-5.9435	5.781648	-0.90991	C	5.282597	-6.94369	-1.9764
C	-5.16211	5.872799	0.254281	C	4.119317	-7.70908	-1.97098
C	-4.01837	6.661628	0.297687	H	-2.33348	-7.2414	1.051587
C	-3.61242	7.400067	-0.82589	H	-4.54634	-6.20374	0.98713
C	-4.46321	7.412273	-1.94484	H	-5.14363	-7.69743	-2.99354
C	-5.60892	6.622458	-1.98556	H	-6.44145	4.316637	-3.11802
C	-2.20502	7.872475	-0.93637	H	-2.92871	-8.76414	-2.92617
C	-1.49022	8.605206	0.034533	H	-7.502	2.12485	-3.22506
C	-0.0907	8.738706	-0.05448	H	-6.66813	-3.1316	-3.11015
C	0.627258	8.112878	-1.09437	H	-5.26734	-5.14248	-2.99907
C	-0.10904	7.521419	-2.1295	H	-5.38834	5.233109	1.101668
C	-1.48827	7.413114	-2.05458	H	-7.8011	-2.00954	0.868764
C	-2.23272	9.315062	1.138877	H	-3.38913	6.643492	1.177731
C	0.626557	9.512944	1.011811	H	-0.79364	-7.86659	-3.07828
C	2.080629	7.815927	-1.04369	H	-7.93607	0.425735	0.933856
C	2.619856	7.24255	0.119165	H	1.643238	-7.70543	-3.04698
C	3.857267	6.609549	0.095273	H	-4.19583	8.008645	-2.81288
C	4.606943	6.523966	-1.09115	H	-9.01532	0.48084	-3.23083
C	2.88351	7.845644	-2.19441	H	-8.84093	-1.96693	-3.30131
C	4.125866	7.217986	-2.2154	H	-6.22307	6.622213	-2.88163
C	5.717334	5.546663	-1.18326	H	0.419068	7.041556	-2.94716
C	6.496395	5.183253	-0.0703	H	-2.01936	6.866632	-2.82694
C	7.283391	4.035791	-0.0876	H	7.338133	1.555101	0.928637
C	7.324661	3.199497	-1.21765	H	7.59215	-0.8722	0.930598
C	6.656348	3.640952	-2.37248	H	8.688249	-0.77236	-3.22449
C	5.872023	4.787899	-2.356	H	8.465316	1.673902	-3.22198
C	7.835318	1.80923	-1.15876	H	5.665464	-4.46303	-2.88525
C	7.679978	1.063733	0.023283	H	6.730354	-2.26245	-3.03233
C	7.819791	-0.3192	0.024275	H	5.069527	-5.61883	1.136956
C	8.119599	-1.01494	-1.15813	H	3.006962	-6.9343	1.138865
C	8.403979	-0.26273	-2.30819	H	5.908763	-6.93128	-2.86445
C	8.272484	1.12323	-2.30598	H	3.862434	-8.29136	-2.85118
C	7.862197	-2.47542	-1.21637	H	2.022293	7.201141	1.024796
C	8.2138	-3.38022	-0.19335	H	4.192643	6.080756	0.981817
C	7.569827	-4.62803	-0.08573	H	2.511196	8.326869	-3.0944
C	6.590176	-5.01352	-1.02474	H	4.70787	7.22623	-3.13221
C	6.395488	-4.17544	-2.13575	H	6.658474	3.021429	-3.2637
C	7.006389	-2.93468	-2.22622	H	5.284807	5.032958	-3.23529
C	9.241437	-3.00995	0.83462	O	-2.86572	8.760059	2.014539
C	7.983349	-5.58375	1.005328	O	-2.12848	10.6375	0.960711
C	5.625822	-6.13781	-0.87735	O	0.336517	9.451467	2.194488
C	4.822443	-6.21968	0.271602	O	1.591076	10.26689	0.478246
C	3.659311	-6.97998	0.272545	O	9.127607	-3.26857	2.020701
C	3.24637	-7.68739	-0.86942	O	10.27561	-2.38742	0.262897

O	7.261742	-5.95367	1.909701
O	9.237448	-5.99343	0.780479
O	-6.69138	-6.74994	1.812022
O	-10.19	-3.89562	0.134894
C	-6.81413	-7.7468	2.901815
C	-11.3979	-3.58301	0.931017
C	2.526952	11.06061	1.305965
C	11.39277	-1.81987	1.050219
C	9.913118	-6.95673	1.681862
C	-2.77863	11.59912	1.882532
C	-9.0837	-4.39292	0.692211
C	-7.50649	-6.75927	0.760063
O	-8.41941	-7.53672	0.55674
O	-8.95451	-4.7126	1.862167
C	12.24473	-1.15662	-0.02908
H	13.12229	-0.68558	0.425424
H	12.58706	-1.89613	-0.76054
H	11.66938	-0.38811	-0.55513
C	10.86519	-0.78236	2.039857
H	10.23903	-1.24198	2.806402
H	11.71378	-0.29393	2.531279
H	10.28461	-0.01553	1.51781
C	12.15642	-2.95088	1.736349
H	11.55773	-3.41544	2.521329
H	12.4405	-3.71546	1.005392
H	13.07197	-2.55103	2.185992
C	11.30843	-7.05125	1.070431
H	11.25404	-7.39215	0.031286
H	11.80498	-6.07572	1.091119
H	11.91708	-7.76264	1.637936
C	9.978825	-6.40015	3.103042
H	8.991422	-6.36045	3.564511
H	10.62475	-7.04541	3.708848
H	10.4015	-5.39168	3.098164
C	9.188216	-8.29965	1.607239
H	8.182082	-8.2312	2.0248
H	9.11729	-8.63785	0.567702
H	9.751303	-9.04956	2.173665
C	-8.17879	-7.60565	3.574111
H	-8.34739	-6.56775	3.875467
H	-8.98522	-7.90938	2.904947
H	-8.206	-8.23854	4.468139
C	-6.56757	-9.1474	2.343045
H	-6.52244	-9.86376	3.170821

H	-7.36494	-9.45162	1.663011
H	-5.61314	-9.18155	1.807311
C	-5.69252	-7.33468	3.851747
H	-4.71804	-7.40903	3.358023
H	-5.83425	-6.30382	4.192307
H	-5.68658	-7.99201	4.727243
C	-12.339	-3.00418	-0.12228
H	-13.2899	-2.72257	0.341413
H	-11.8989	-2.11459	-0.58448
H	-12.5398	-3.74043	-0.90747
C	-11.0642	-2.53798	1.994599
H	-11.9882	-2.22852	2.495305
H	-10.3799	-2.93591	2.745878
H	-10.6126	-1.65397	1.53329
C	-11.961	-4.87278	1.525761
H	-12.1105	-5.62017	0.73918
H	-11.2923	-5.28813	2.281457
H	-12.9313	-4.66497	1.99035
C	-2.26302	11.4027	3.30707
H	-2.62708	12.2242	3.934144
H	-2.60731	10.45888	3.731604
H	-1.16971	11.41313	3.321715
C	-2.32662	12.94473	1.321465
H	-2.64705	13.05825	0.280581
H	-2.76179	13.7592	1.90953
H	-1.23602	13.03188	1.361127
C	-4.29477	11.44136	1.777288
H	-4.61647	11.53322	0.734141
H	-4.62089	10.4736	2.162485
H	-4.78435	12.23136	2.357295
C	1.754659	12.15831	2.035
H	1.151581	12.73439	1.325132
H	1.098958	11.74105	2.800747
H	2.462305	12.84236	2.516215
C	3.289605	10.14578	2.262775
H	3.790755	9.345382	1.709906
H	4.054105	10.73243	2.783892
H	2.626165	9.702616	3.007389
C	3.464737	11.65193	0.256542
H	2.908427	12.2729	-0.45341
H	4.223223	12.27425	0.742291
H	3.970512	10.85626	-0.29972
H	1.664773	-8.77403	1.118062
H	-0.78135	-8.93461	1.086457

H	-7.64554	4.708875	0.989874	H	6.453756	5.781878	0.835048
H	7.839433	3.762702	0.804528	H	-8.7256	2.512253	0.880601
